# Supplementary material for: α-Methylacyl-CoA Racemase from Mycobacterium tuberculosis—Detailed Kinetic and Structural Characterization of the Active Site
Source: Biomolecules. 2024 Mar 2;14(3):299. doi: 10.3390/biom14030299 (PMC10967716; doi:10.3390/biom14030299)
Supplement: Supplementary file 1 [file biomolecules-14-00299-s001.zip › biomolecules-2861071-supplementary.pdf]

α-Methylacyl-CoA Racemase from *Mycobacterium tuberculosis*—Detailed Kinetic and Structural Characterization of the Active site

Otsile O. Mojanaga, Timothy J. Woodman, Matthew D. Lloyd * and K. Ravi Acharya *

Department of Life Sciences, University of Bath, Claverton Down, Bath BA2 7AY, UK; oom21@bath.ac.uk (O.O.M.); tw226@bath.ac.uk (T.J.W.)

* Correspondence: m.d.lloyd@bath.ac.uk (M.D.L.); bsskra@bath.ac.uk (K.R.A.); Tel.: +44-(0)1225-386786 (M.D.L.); Tel.: +44-(0)1225-386238 (K.R.A.)

3 ....Scheme S1. Colorimetric reaction catalysed by MCR
4 ....Figure S1. Deconvoluted intact protein mass spectra for wild-type MCR and 3 mutants
6 .... Figure S2. Purification of H126A MCR
9 ....Figure S3. Purification of D156A MCR
12 .... Figure S4. Purification of E241A MCR
14 ....Figure S5. Alignment of determined and published MCR sequences
15 ....Figure S6. Dynamic light scattering analyses of wild-type MCR
15 ...... Figure S7. Standard curve from the analytical chromatography of Wild-type MCR using a Superdex-200 column
17 ....Figure S8. Kinetic parameters for wild-type MCR as determined by the colorimetric assay
21 ....Figure S9. Kinetic parameters for H126A MCR as determined by the colorimetric assay
26 ....Figure S10. Kinetic parameters for D156A MCR as determined by the colorimetric assay
29 ....Figure S11. Kinetic parameters for E241A MCR as determined by the colorimetric assay
34 .... Table S1. Apparent kinetic parameters of wild-type MCR and its 3 mutants
34 ....References

Scheme S1. Colorimetric reaction catalysed by MCR

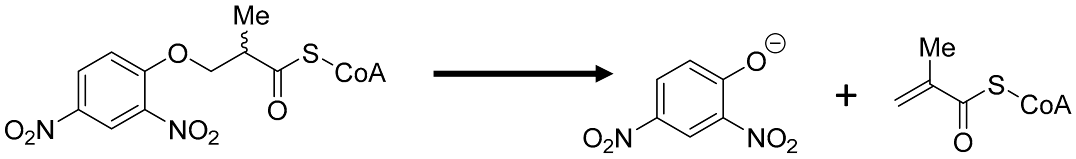

1 **2** **3**

Scheme S1: The colorimetric assay reaction. The colourless substrate (3-(2,4-dinitrophenoxy)-2-methylpropanoyl-CoA) **1** undergoes an elimination to form 2,4-dinitrophenolate **2** (yellow) and an unsaturated product (2-methylpropanoyl-CoA) **3**. The formation of 2,4-dinitrophenolate 2 over time can be monitored at 354 nm [1].

Figure S1. Deconvoluted intact protein mass spectra for wild-type MCR and 3 mutants

Wild-type MCR

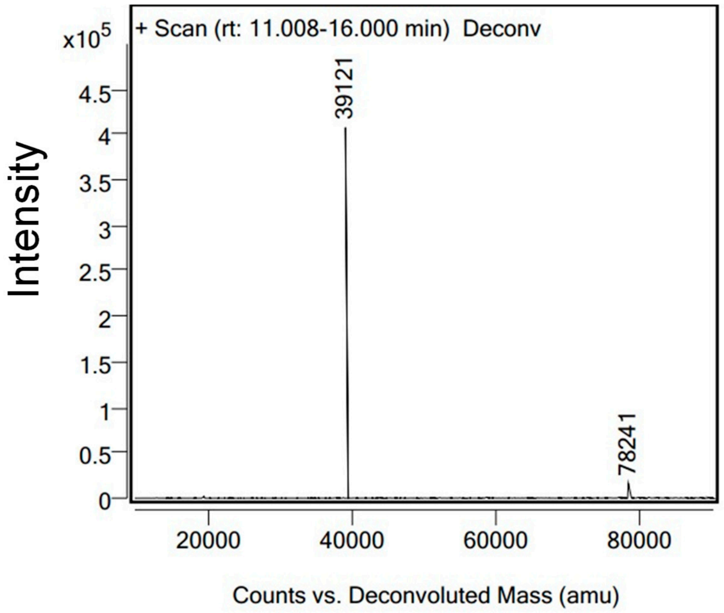

H126A mutant

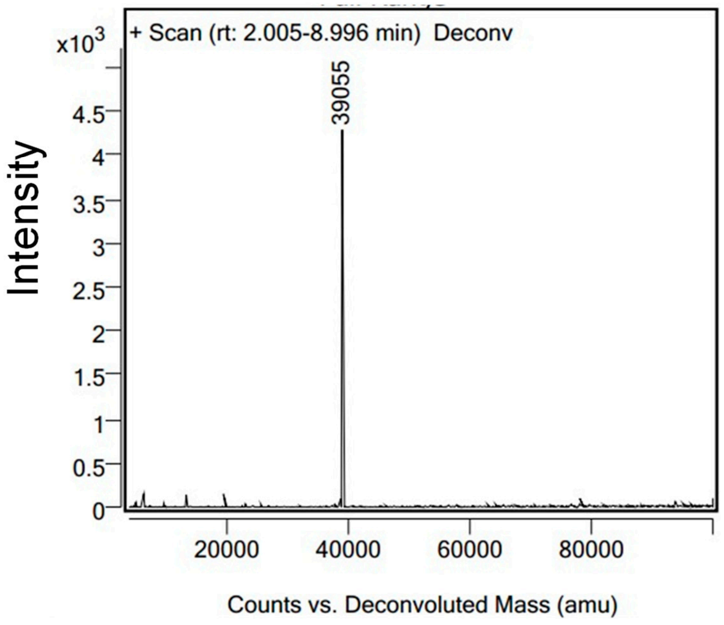

D156A mutant

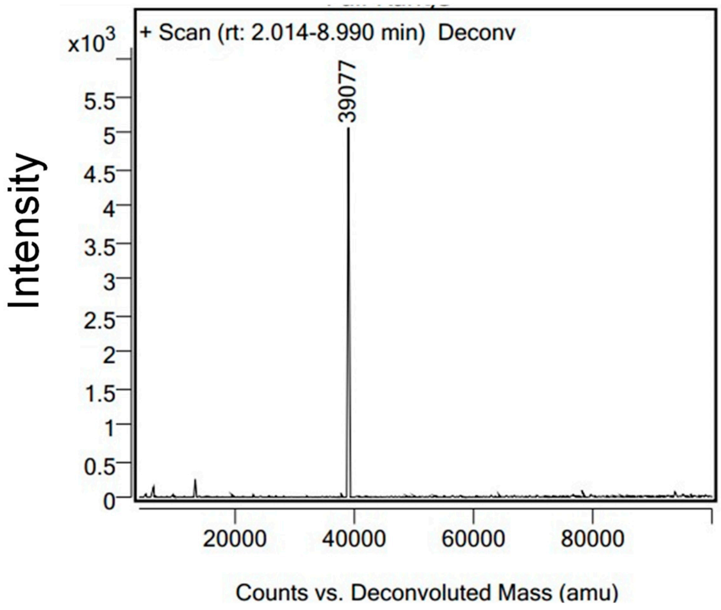

E241A mutant

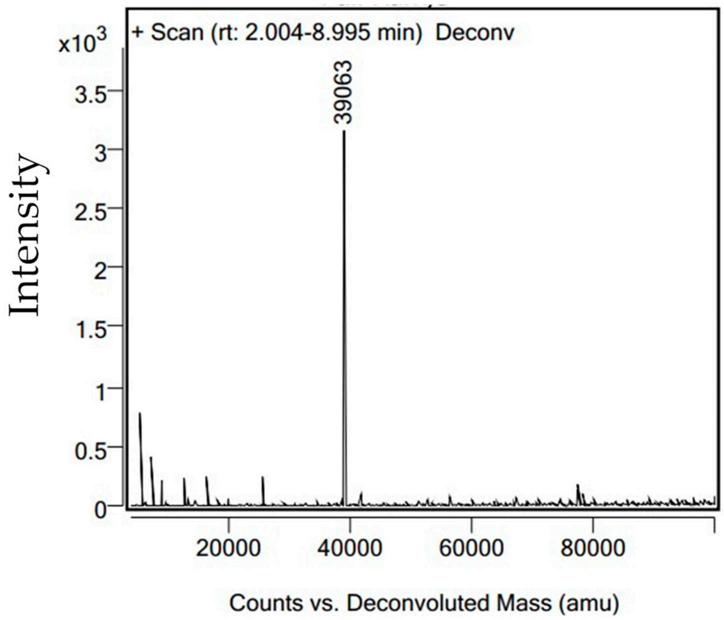

Figure S1. Deconvoluted LC-MS/MS mass spectra of MCR WT and the 3 mutants. Positive mode Electrospray ionisation mode on the Agilent QTOF 6545 system coupled to a HPLC Agilent 1260 Infinity II Quat pump was used to collect intact protein mass spectrometry data. Data was deconvoluted using the Mass Hunter BioConfirm 10.0 software. Wild-type MCR and 3 mutants had molecular weights that were 437 Da higher than expected due to an additional Met residue at the N-terminus and a short extension of amino acids (GSGC) at the C-terminus. Determined molecular weights were 39 121 Da, 39 055 Da, 39 077 Da, and 39 063 Da for wild-type MCR and the H126A, D156A, and E241A mutants, respectively.

Figure S2. Purification of H126A MCR

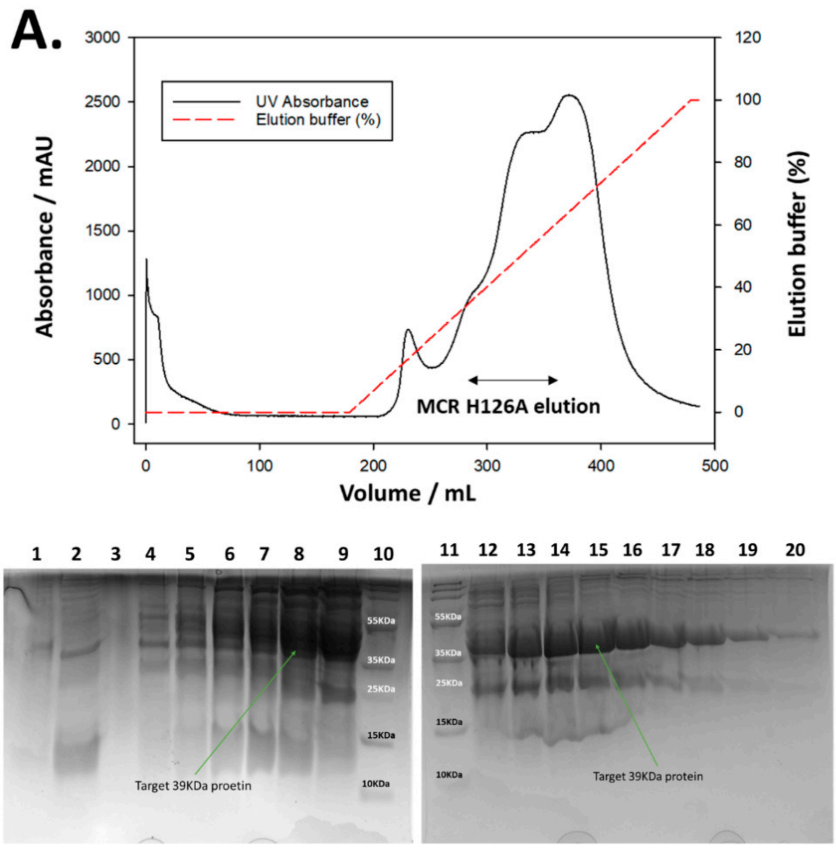

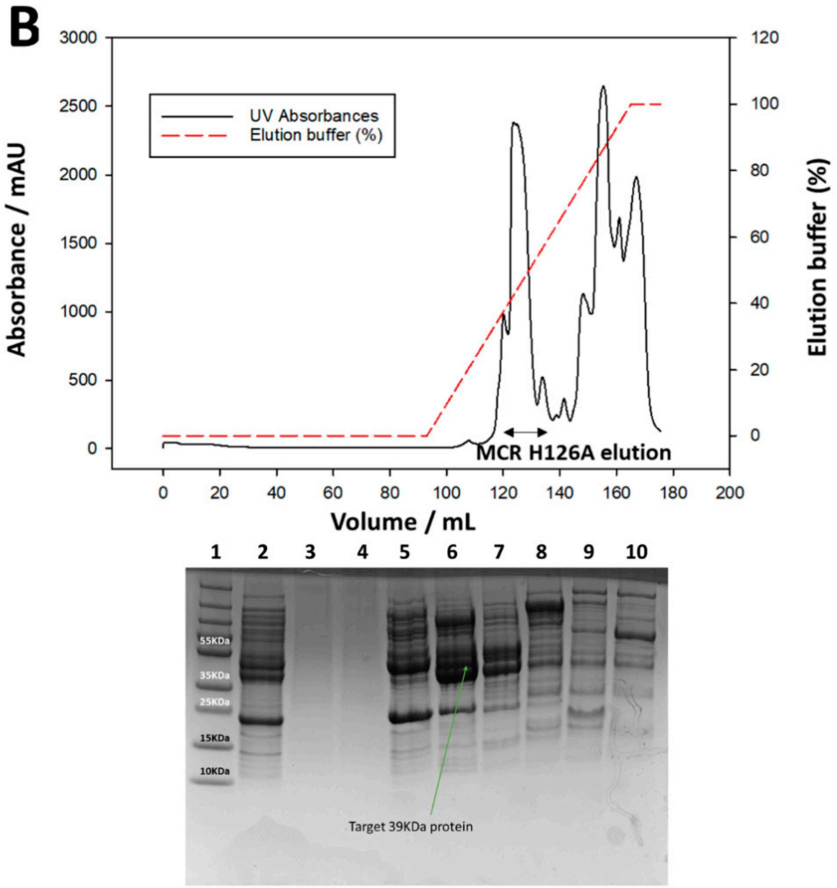
B
Absorbance / mAU
Elution buffer (%)
UV Absorbances
Elution buffer (%)
MCR H126A elution
Volume / mL
1 2 3 4 5 6 7 8 9 10
55KDa
35KDa
25KDa
15KDa
10KDa
Target 39KDa protein

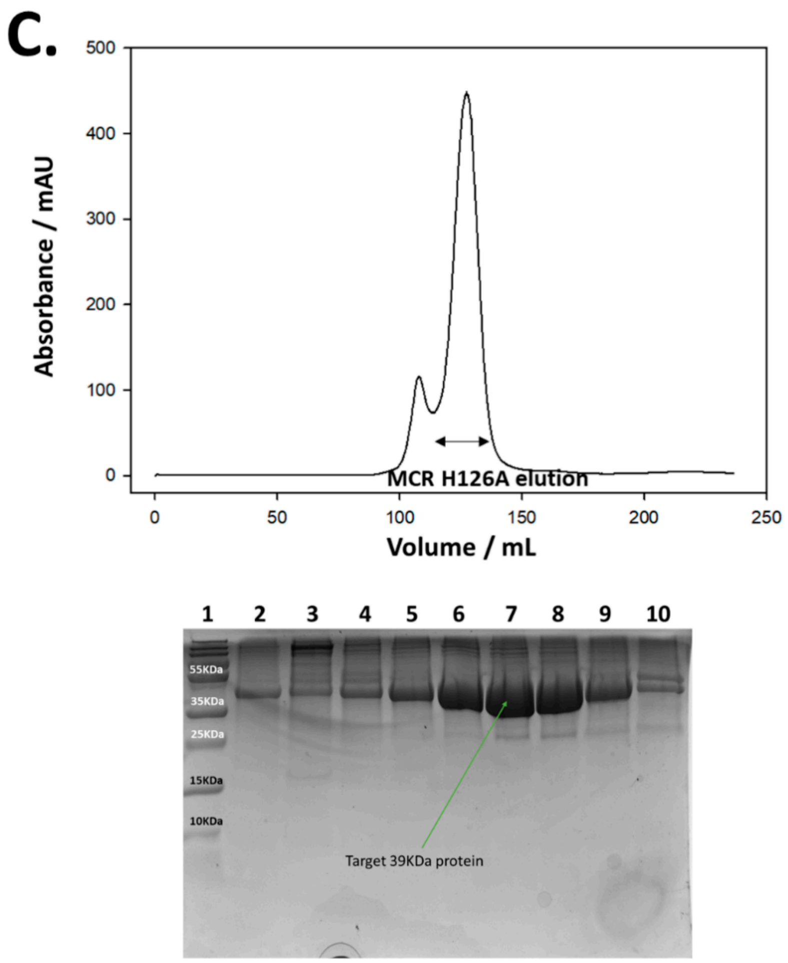

Figure S2. Chromatograms and gels from the purification of recombinant MCR H126A. Chromatography traces show the absorbance at 280 nm and elution gradients are shown where appropriate. SDS-PAGE analysis was performed using 12 % Tris-glycine gels. **A**. DEAE anion-exchange chromatography of the cell lysate with MCR eluting between 295-415 mL (0.23 – 0.46 mM NaCl). Fractions shown on SDS-PAGE analysis are as follows: 1. Load; 2. Flow-Through; 3. Wash; 4 to 9, elution fractions A9 to A14; 10 and 11, 10-180 kDa markers; 12 to 20, elution fractions A15 to B8. **B**. RESOURCE-Q anion-exchange chromatography with MCR eluting between 125-132 mL (0.27 – 0.32 mM NaCl). Fractions (3 mL) shown on SDS-PAGE analysis are as follows: 1. 10-180 kDa markers; 2. Load; 3. Flow-through; 4. Wash; 5 to 10, eluted fractions A10 to A15. **C**. Sephacryl-100 size-exclusion chromatography with MCR eluting between 119 and 139 mL. Fractions (2 mL) shown on SDS-PAGE analysis are as follows: 1. 10-180 kDa markers; 2 to 10, eluted fractions C2 to D12 (106 to 131 mL).

Figure S3. Purification of D156A MCR

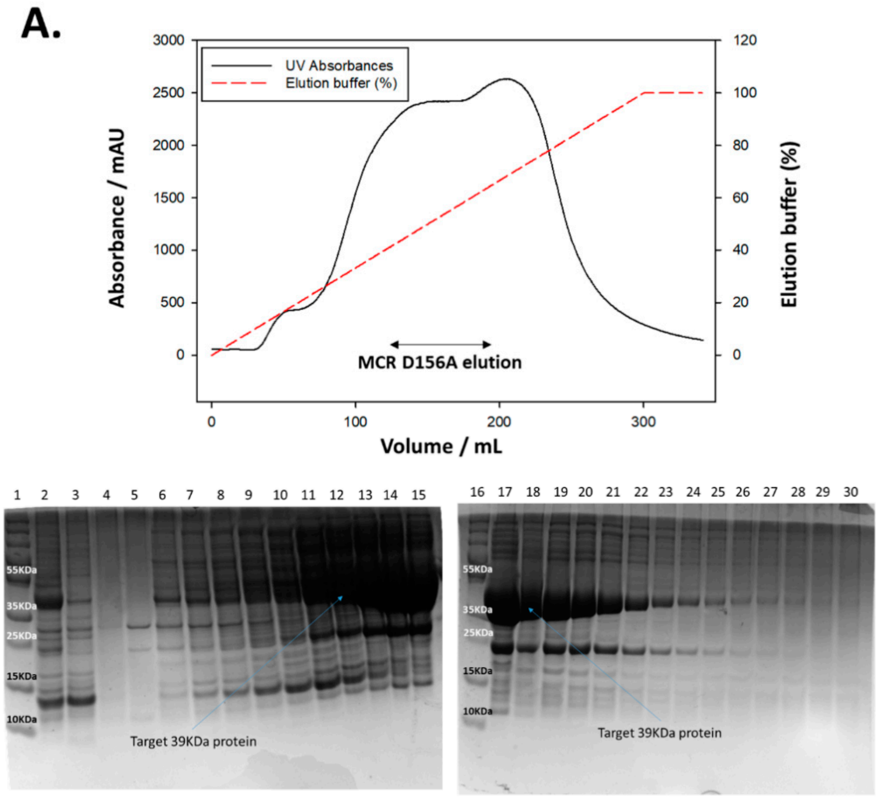

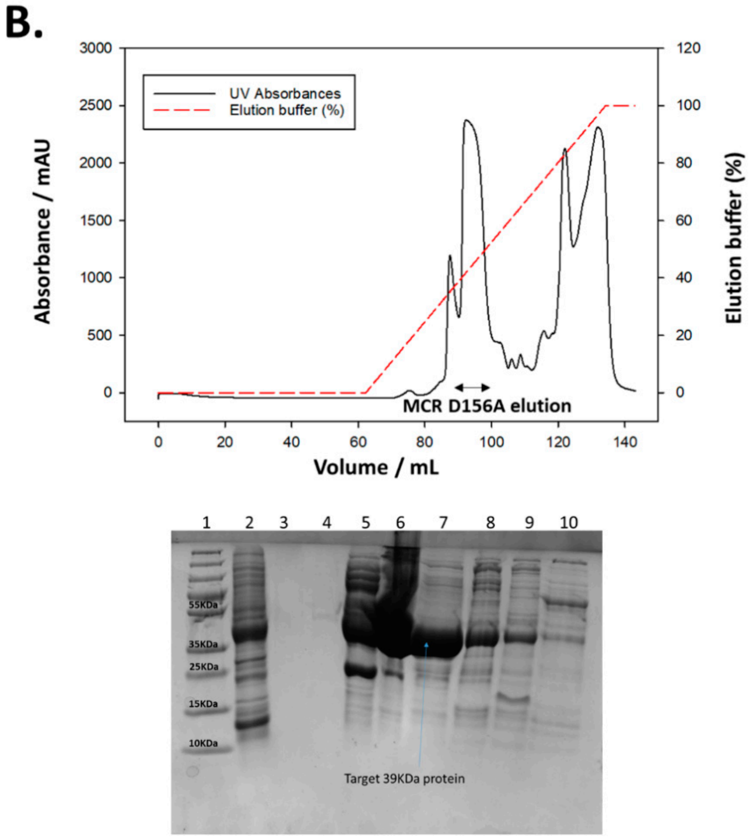
B.
UV Absorbances
Elution buffer (%)
Absorbance / mAU
Elution buffer (%)
Volume / mL
MCR D156A elution
1 2 3 4 5 6 7 8 9 10
55KDa
35KDa
25KDa
15KDa
10KDa
Target 39KDa protein

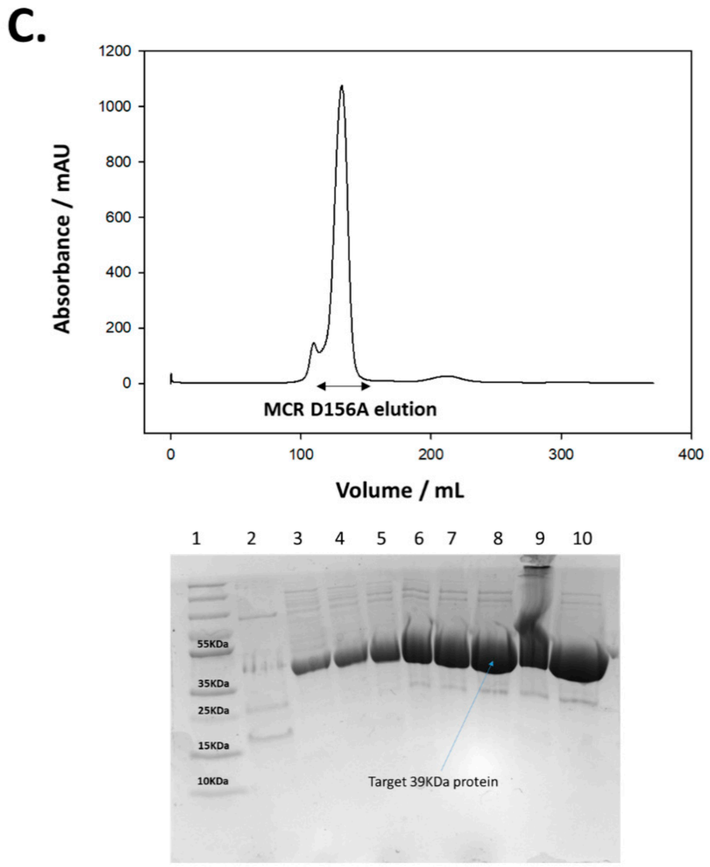

Figure S3. Chromatograms and gels from the purification of recombinant MCR D156A. Chromatography traces show the absorbance at 280 nm and elution gradients are shown where appropriate. SDS-PAGE analysis was performed using 12 % Tris-glycine gels. **A.** DEAE anion-exchange chromatography of the cell lysate with MCR eluting between 135-225 mL (0.27 – 0.43 mM NaCl). Fractions shown on SDS-PAGE analysis are as follows: 1. 10-180 kDa markers; 2. Load; 3. Flow-Through; 4. Wash; 5 to 10, elution fractions A8 to B7; 16, 10-180 kDa markers; 17 to 30, elution fractions B6 to C8. **B**. RESOURCE-Q anion-exchange chromatography with MCR eluting between 92-101 mL (0.25 – 0.35 mM NaCl). Fractions (3 mL) shown on SDS-PAGE analysis are as follows: 1. 10-180 kDa markers; 2. Load; 3. Flow-through; 4. Wash; 5 to 15, eluted fractions A11 to B9. **C.** Sephacryl-100 size-exclusion chromatography with MCR eluting between 118 and 140 mL. Fractions (2 mL) shown on SDS-PAGE analysis are as follows: 1. 10-180 kDa markers; 2 to 10, eluted fractions C6 to D12 (114 to 130 mL).

Figure S4. Purification of E241A MCR

A.

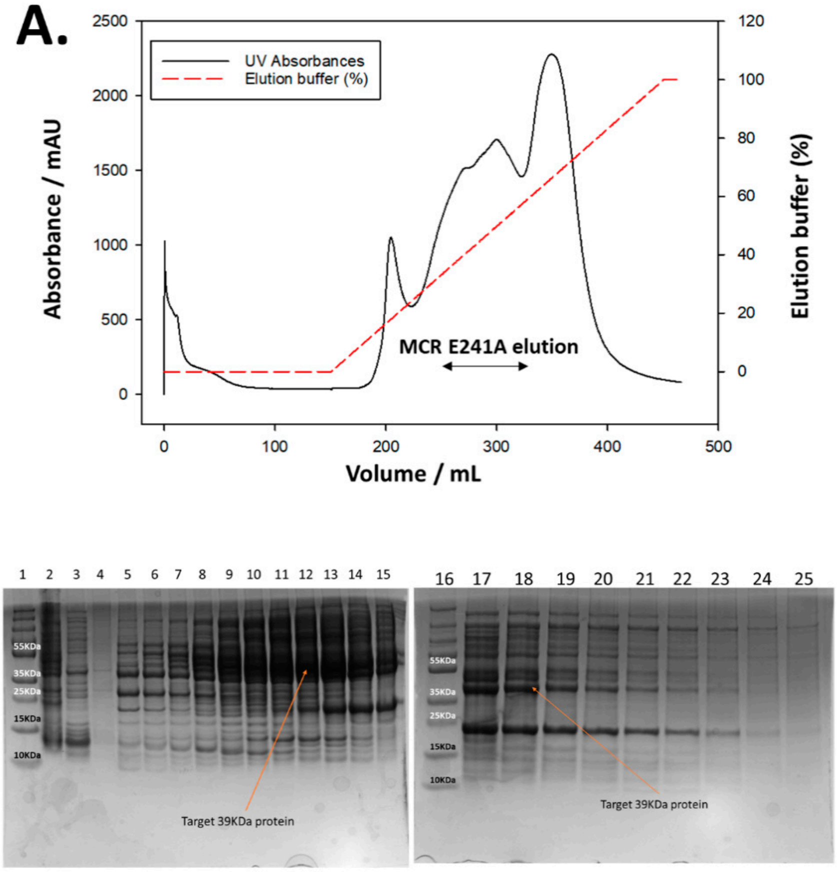

B.

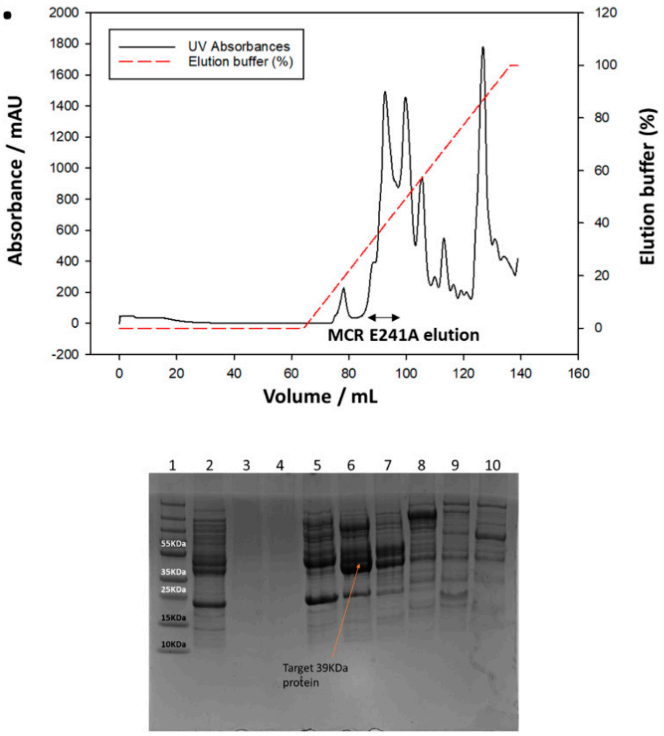

C.

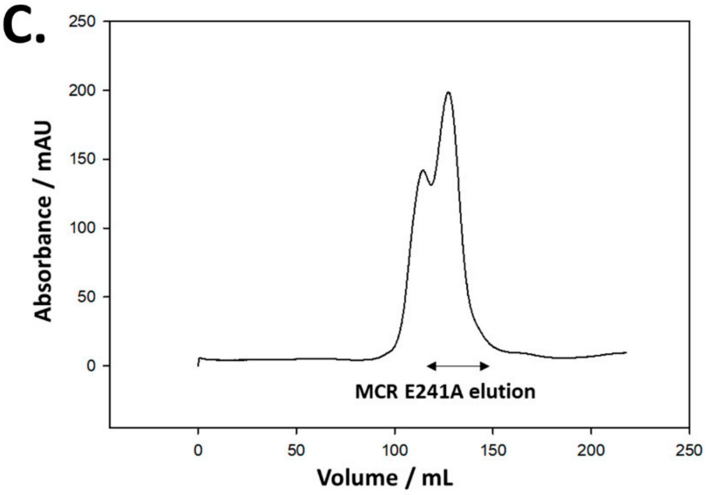

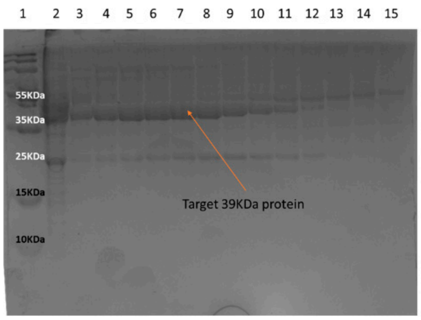

Figure S4. Chromatograms and gels from the purification of recombinant MCR E241A. Chromatography traces show the absorbance at 280 nm and elution gradients are shown where appropriate. SDS-PAGE analysis was performed using 12 % Tris-glycine gels. **A**. DEAE anion-exchange chromatography of the cell lysate with MCR eluting between 285-348 mL (0.27 – 0.40 mM NaCl). Fractions shown on SDS-PAGE analysis are as follows: 1. 10-180 kDa markers; 2. Load; 3. Flow-Through; 4. Wash; 5 to 15, elution fractions A8 to B7; 16, 10-180 kDa markers; 17 to 30, elution fractions B6 to C3. **B.** RESOURCE-Q anion-exchange chromatography with MCR eluting between 98 - 102 mL (0.28 – 0.32 mM NaCl). Fractions (3 mL) shown on SDS-PAGE analysis are as follows: 1. 10-180 kDa markers; 2. Load; 3. Flow-through; 4. Wash; 5 to 10, eluted fractions A11 to B9. C. Sephacryl-100 size-exclusion chromatography with MCR eluting between 121 and 133 mL. Fractions (2 mL) shown on SDS-PAGE analysis are as follows: 1. 10-180 kDa markers; 2 to 10, eluted fractions C8 to D5 (119 to 145 mL).

Figure S5. Alignment of determined and published MCR sequences

```
Published MCR     -MAGPLSGLRVVELAGIGPGPHAAMILGDLGADVVRIDRPSSVDGISRDAMLRNRRIVTA  59
Translated MCR    MMAGPLSGLRVVELAGIGPGPHAAMILGDLGADVVRIDRPSSVDGISRDAMLRNRRIVTA  60

Published MCR     DLKSDQGLELALKLIAKADVLIEGYRPGVTERLGLGPEECAKVNDRLIYARMTGWGQTGP  119
Translated MCR    DLKSDQGLELALKLIAKADVLIEGYRPGVTERLGLGPEECAKVNDRLIYARMTGWGQTGP  120

Published MCR     RSQQAGHDINYISLNGILHAIGRGDERPVPPLNLVGDFGGGSMFLLVGILAALWERQSSG  179
Translated MCR    RSQQAGHDINYISLNGILHAIGRGDERPVPPLNLVGDFGGGSMFLLVGILAALWERQSSG  180

Published MCR     KGQVVDAAMVDGSSVLIQMMWAMRATGMWTDTRGANMLDGGAPYYDTYECADGRYVAVGA  239
Translated MCR    KGQVVDAAMVDGSSVLIQMMWAMRATGMWTDTRGANMLDGGAPYYDTYECADGRYVAVGA  240

Published MCR     IEPQFYAAMLAGLGLDAAELPPQNDRARWPELRALLTEAFASHDRDHWGAVFANSDACVT  299
Translated MCR    IEPQFYAAMLAGLGLDAAELPPQNDRARWPELRALLTEAFASHDRDHWGAVFANSDACVT  300

Published MCR     PVLAFGEVHNEPHIIERNTFYEANGGWQPMPAPRFSRTASSQPRPPAATIDIEAVLTDWD  359
Translated MCR    PVLAFGEVHNEPHIIERNTFYEANGGWQPMPAPRFSRTASSQPRPPAATIDIEAVLTDWD  360

Published MCR     G---- 360
Translated MCR    GGSGC 365
```

Figure S5 Sequence alignment of the wild-type MCR from *M. tuberculosis* (Uniprot O06543) (Published MCR) and the over-produced recombinant MCR from *M. tuberculosis* (this experiment). The bold and underlined residues show the additional 1 N-terminal and 4 C-terminal residues on the recombinant MCR. The protein sequence for this experiment was obtained by translation of the corresponding DNA sequence and confirmed by mass spectrometric analysis (Figure S1).

Figure S6. Dynamic light scattering analyses of wild-type MCR

A.

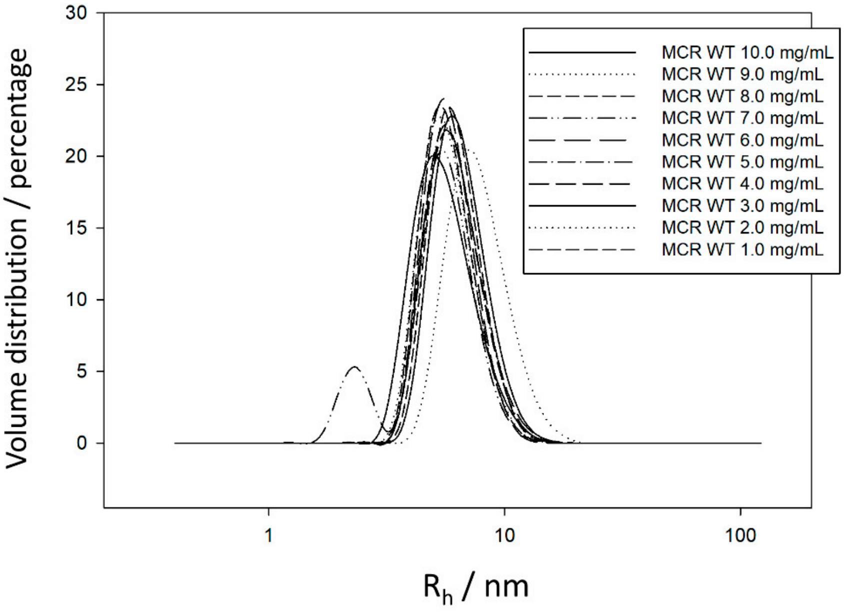

B.

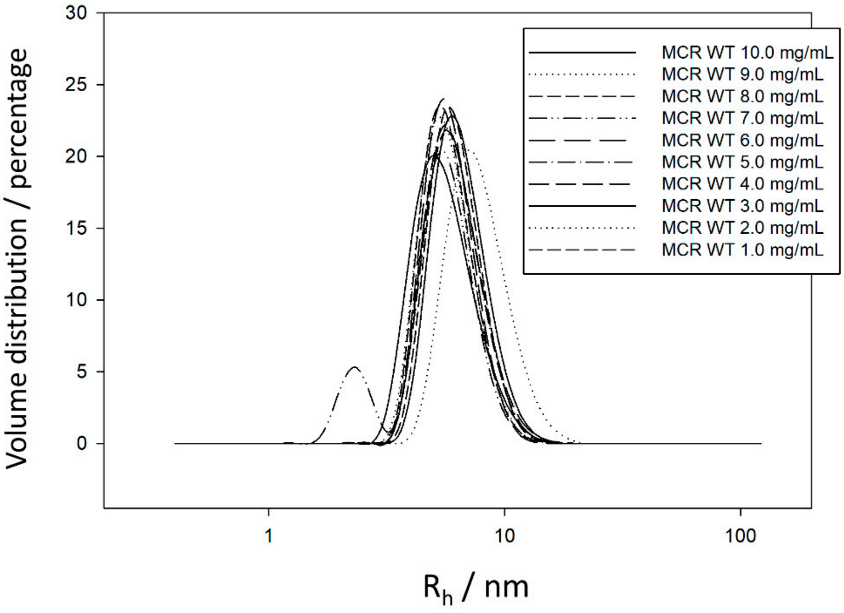

Figure S6. Dynamic light scattering analyses of wild-type MCR. Data for 10 mg/mL of wild-type MCR was measured using a Zetasizer Nano S system. Protein stocks between 1 to 10 mg/mL in 10 mM potassium phosphate, pH 8.8 buffer were used in dynamic laser light scattering analyses. Distributions of volume (**A**) and intensity (**B**) against thermodynamic radii (R_h) are shown. The results suggest that the purified recombinant enzyme is monodispersed.

Figure S7. Standard curve from the analytical chromatography of Wild-type MCR using a Superdex-200 column

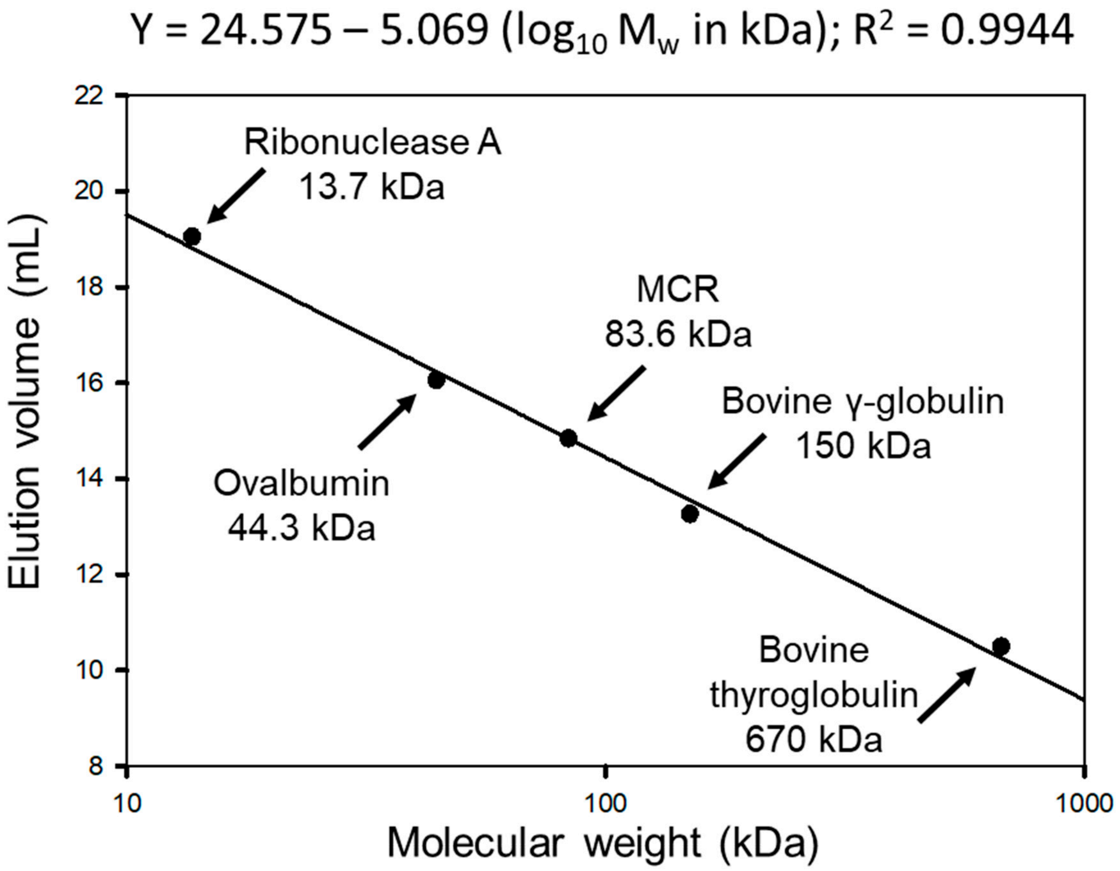

Figure S7. A standard curve from analytical size exclusion chromatography of recombinant wild-type MCR and 4 standard proteins. 1.64 mg of wild-type MCR was analysed using a Superdex-200 10/300 column. 24 mg of a 4-protein standard was used to calibrate the column and elution was monitored using absorbance at 280 nm. Elution volumes for the protein standards were as follows: Bovine thyroglobulin (670 kDa; 10.5 mL); Bovine γ-globulin (150 kDa; 13.3 mL); Ovalbumin (44.3 kDa; 16.1 mL); Bovine ribonuclease A (13.7 kDa; 19.1 mL). The protein standards were used to generate the standard curve with the equation shown. An elution volume of 14.83 mL was obtained for wild-type MCR, corresponding to a molecular weight of 89.0 KDa, consistent with the formation of a dimer.

Figure S8. Kinetic parameters for wild-type MCR as determined by the colorimetric assay

Parameters

	Value	±Std. Error	95% Conf. Interval
Vmax (μmol.min^{-1}.mg^{-1})	157.1370	12.7554	130.6834 to 183.5905
Km (μM)	96.1983	14.4384	66.2543 to 126.1422

Goodness of Fit

Degrees of Freedom	22
AICc	88.088
R^2	0.965
Sum of Squares	698.105
Sy.x	5.633
Runs Test p Value	0.501

Data

Number of x values	8
Number of replicates	3
Total number of values	24
Number of missing values	0

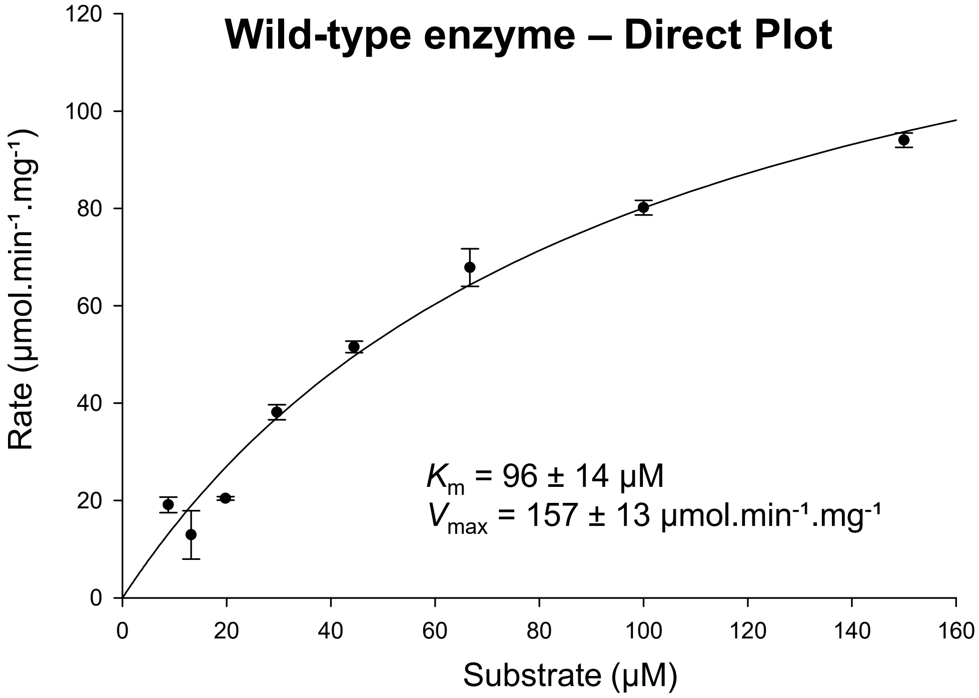
Wild-type enzyme – Direct Plot
Rate (µmol.min⁻¹.mg⁻¹)
Substrate (µM)
K_m = 96 ± 14 µM
V_{max} = 157 ± 13 µmol.min⁻¹.mg⁻¹
0
20
40
60
80
100
120
140
160

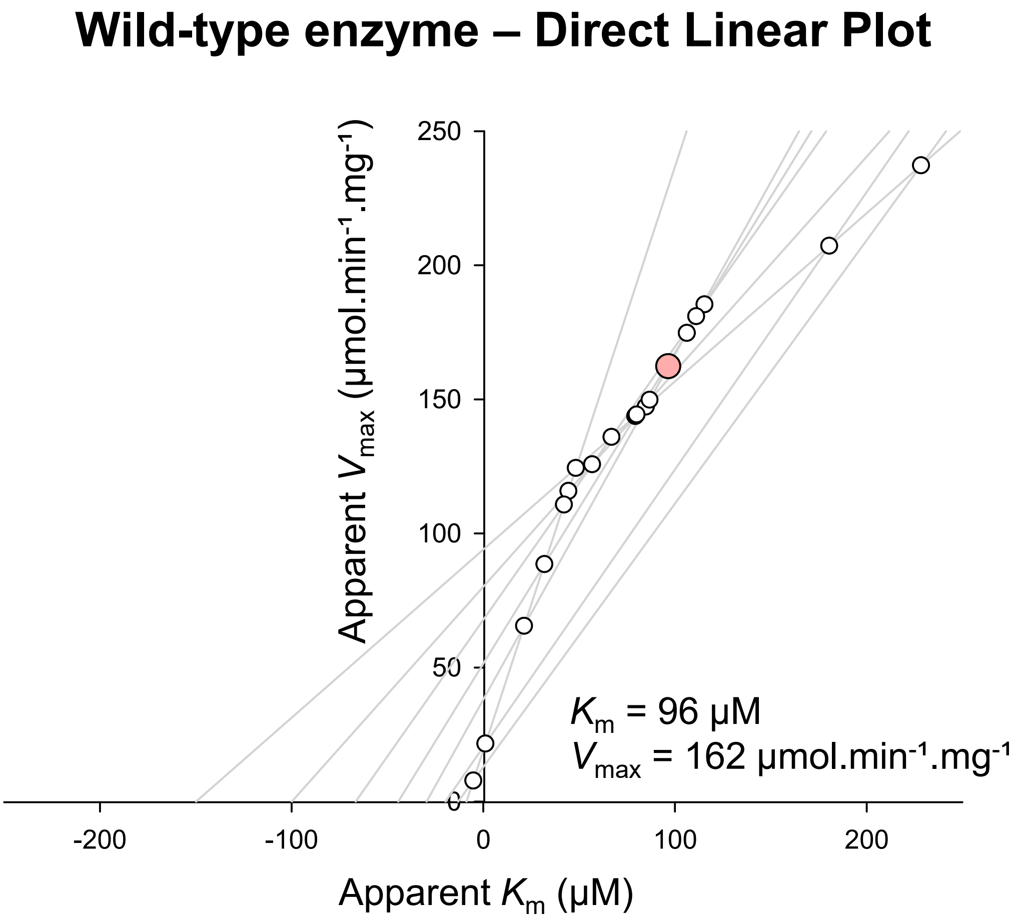
Wild-type enzyme – Direct Linear Plot
Apparent V_{max} (µmol.min⁻¹.mg⁻¹)
Apparent K_m (µM)
K_m = 96 µM
V_{max} = 162 µmol.min⁻¹.mg⁻¹
250
200
150
100
50
0
-200
-100
0
100
200

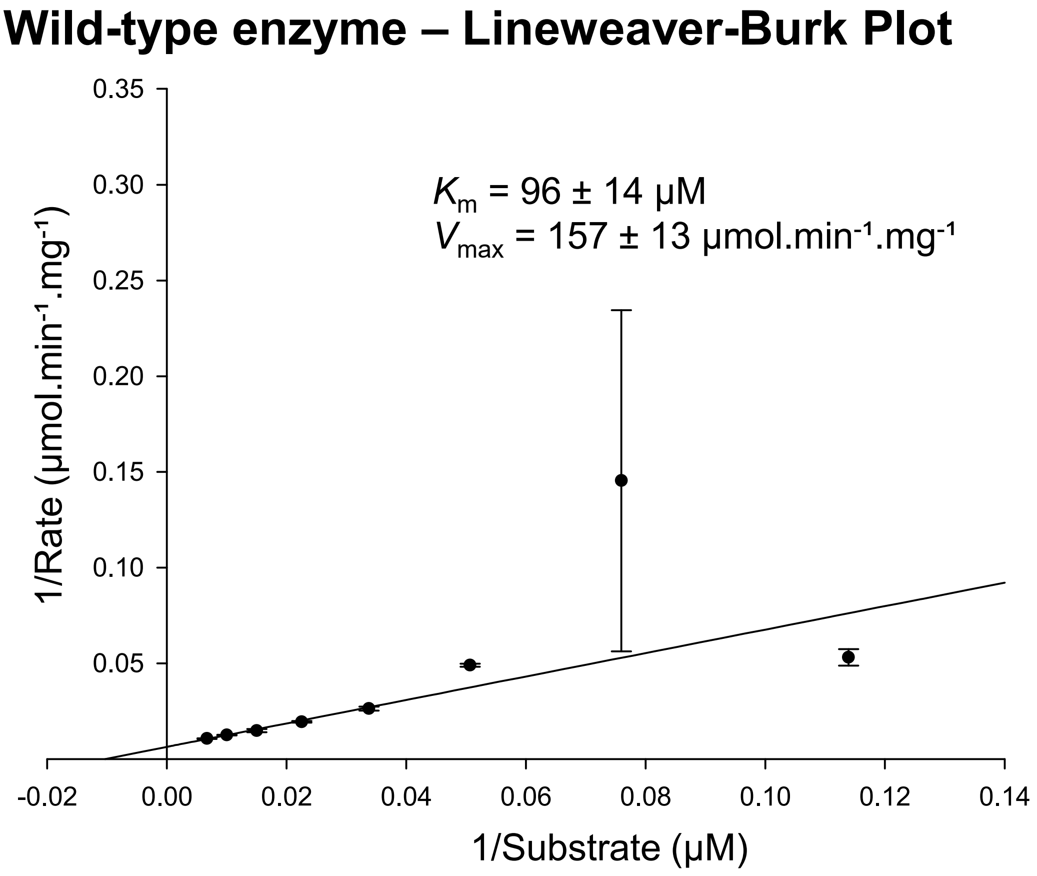
Wild-type enzyme – Lineweaver-Burk Plot
K_m = 96 ± 14 μM
V_{max} = 157 ± 13 μmol.min⁻¹.mg⁻¹
1/Rate (μmol.min⁻¹.mg⁻¹)
1/Substrate (μM)
0.35
0.30
0.25
0.20
0.15
0.10
0.05
-0.02
0.00
0.02
0.04
0.06
0.08
0.10
0.12
0.14

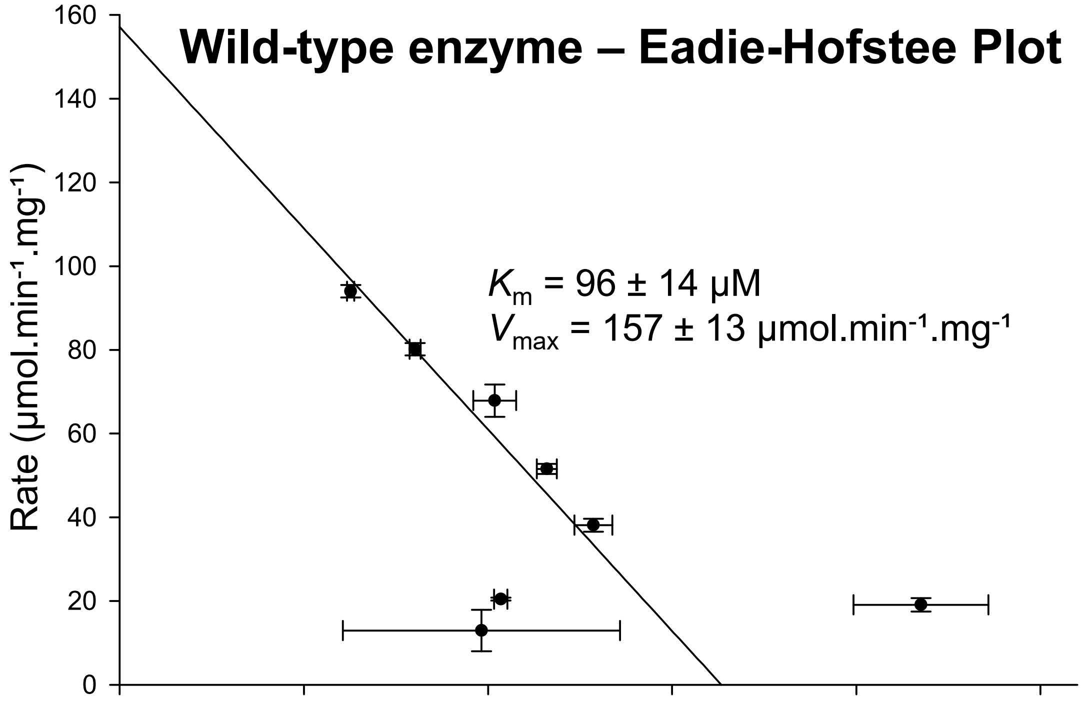
Wild-type enzyme – Eadie-Hofstee Plot
K_m = 96 ± 14 μM
V_{max} = 157 ± 13 μmol.min⁻¹.mg⁻¹
Rate (μmol.min⁻¹.mg⁻¹)
Rate (μmol.min⁻¹.mg⁻¹) / Substrate (μM)
160
140
120
100
80
60
40
20
0
0.0
0.5
1.0
1.5
2.0
2.5

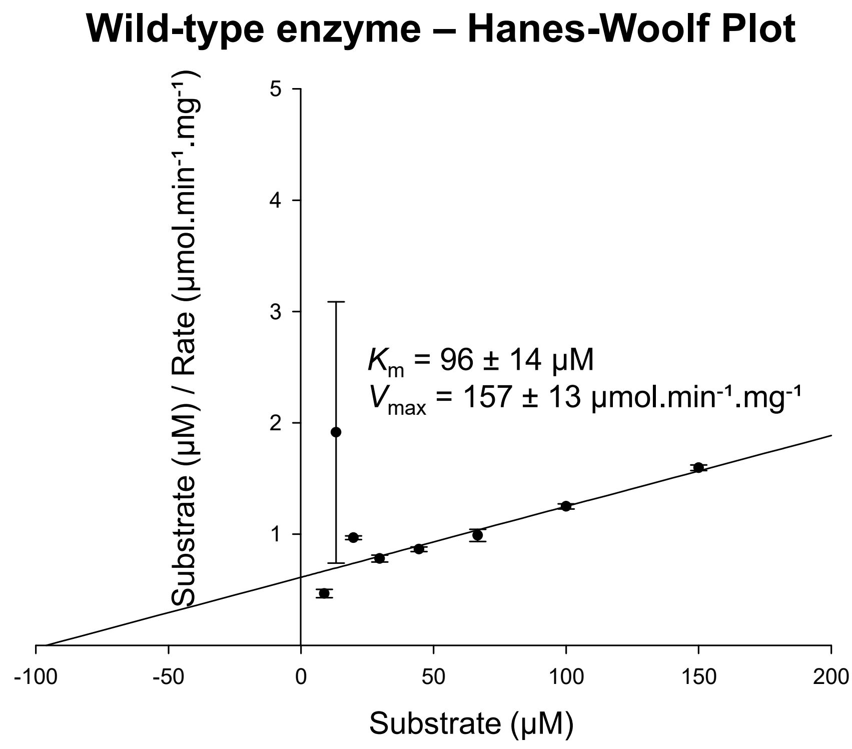

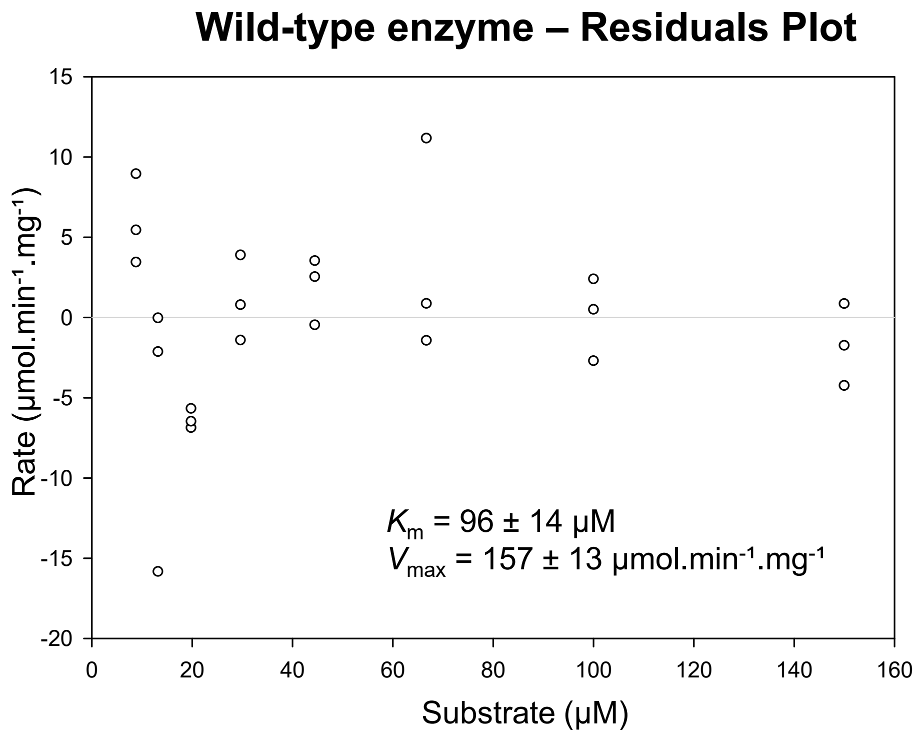

Figure S8. Kinetic data for wild-type MCR. Using optimized enzyme concentrations, dilutions of the colorimetric substrate were separately mixed with 0.122 µg/mL wild-type MCR and A_{354} data was recorded. This absorbance data was used to determine rates (in µmol.min^{-1}.mg^{-1}). Data for 3 dependent repeats was measured over 10 min. Plotted data are means ± SD.

Figure S9. Kinetic parameters for H126A MCR as determined by the colorimetric assay

Parameters

	Value	± Std. Error	95% Conf. Interval
V_{max} (μmol.min^{-1}.mg^{-1})	11.0428	0.4488	10.1120 to 11.9736
K_m (μM)	63.3629	5.4753	52.0076 to 74.7182

Goodness of Fit

Degrees of Freedom	22
AICc	-51.595
R^2	0.982
Sum of Squares	2.071
Sy.x	0.307
Runs Test p Value	0.501

Data

Number of x values	8
Number of replicates	3
Total number of values	24
Number of missing values	0

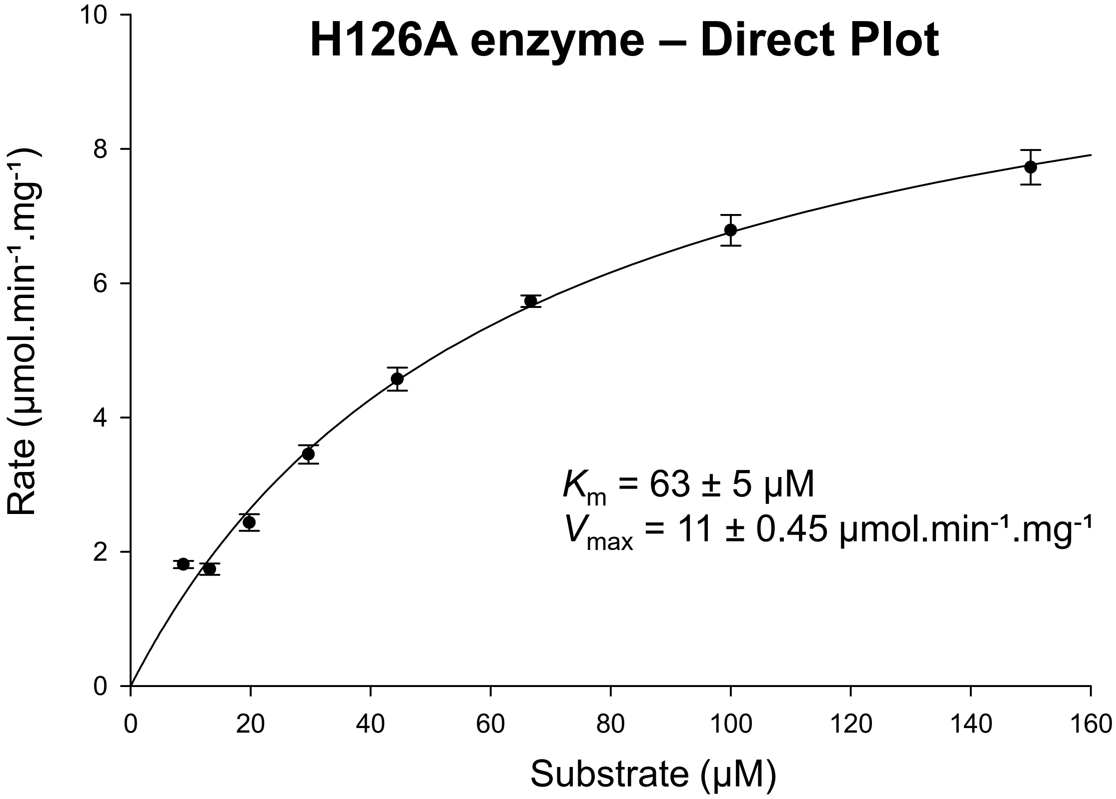
H126A enzyme – Direct Plot
Rate (µmol.min⁻¹.mg⁻¹)
Substrate (µM)
K_m = 63 ± 5 µM
V_{max} = 11 ± 0.45 µmol.min⁻¹.mg⁻¹
0
2
4
6
8
10
0
20
40
60
80
100
120
140
160

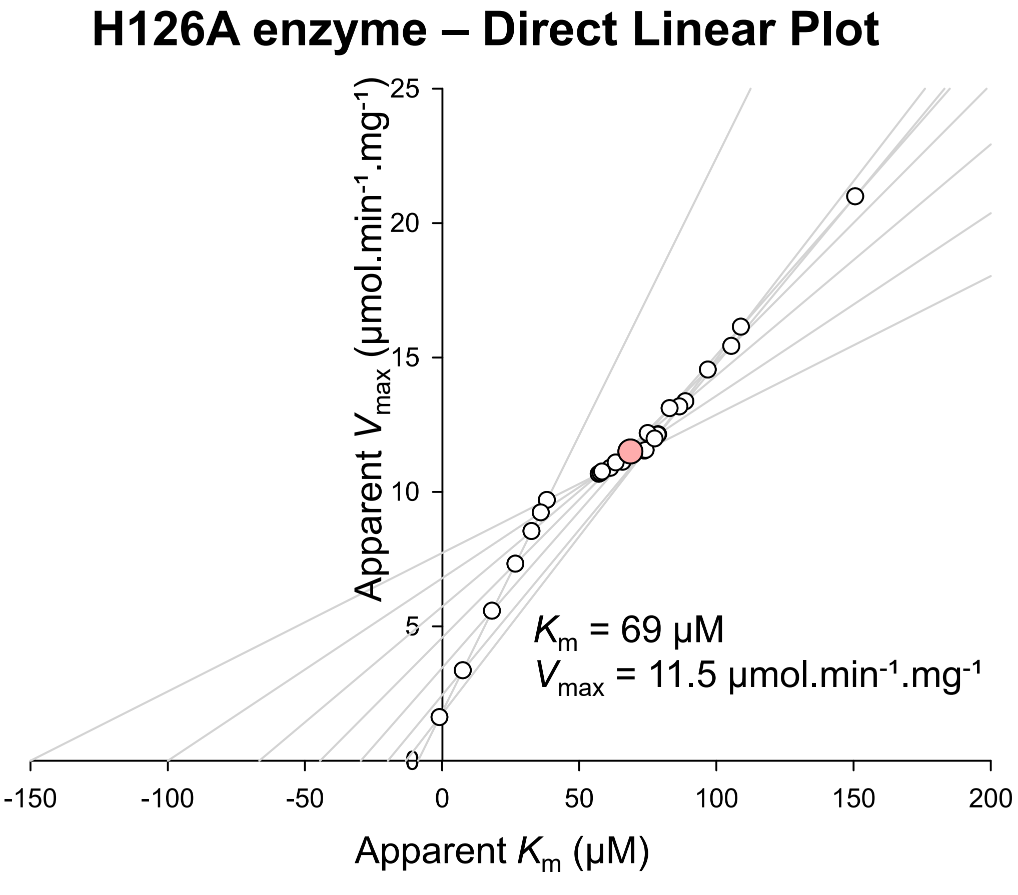
H126A enzyme – Direct Linear Plot
Apparent V_{max} (µmol.min⁻¹.mg⁻¹)
Apparent K_m (µM)
K_m = 69 µM
V_{max} = 11.5 µmol.min⁻¹.mg⁻¹
0
5
10
15
20
25
-150
-100
-50
0
50
100
150
200

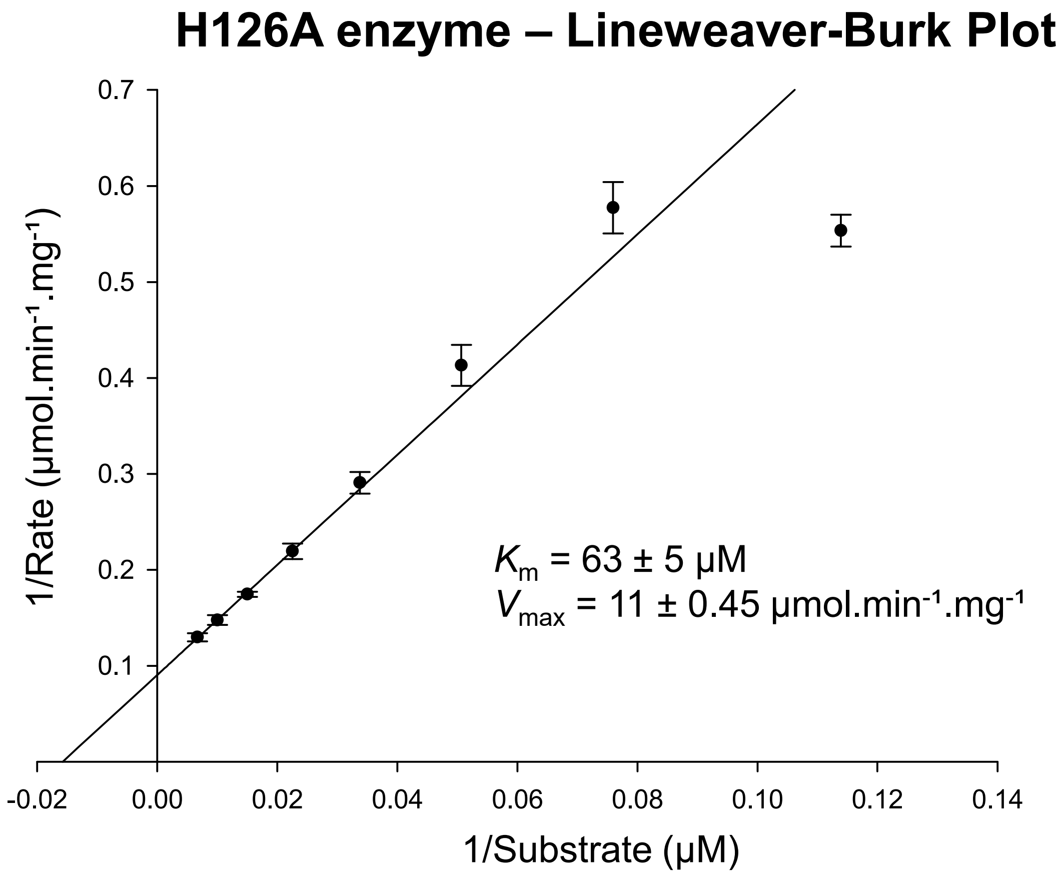

H126A enzyme – Lineweaver-Burk Plot
0.7
0.6
0.5
0.4
0.3
0.2
0.1
1/Rate (µmol.min⁻¹.mg⁻¹)
K_m = 63 ± 5 µM
V_{max} = 11 ± 0.45 µmol.min⁻¹.mg⁻¹
-0.02
0.00
0.02
0.04
0.06
0.08
0.10
0.12
0.14
1/Substrate (µM)

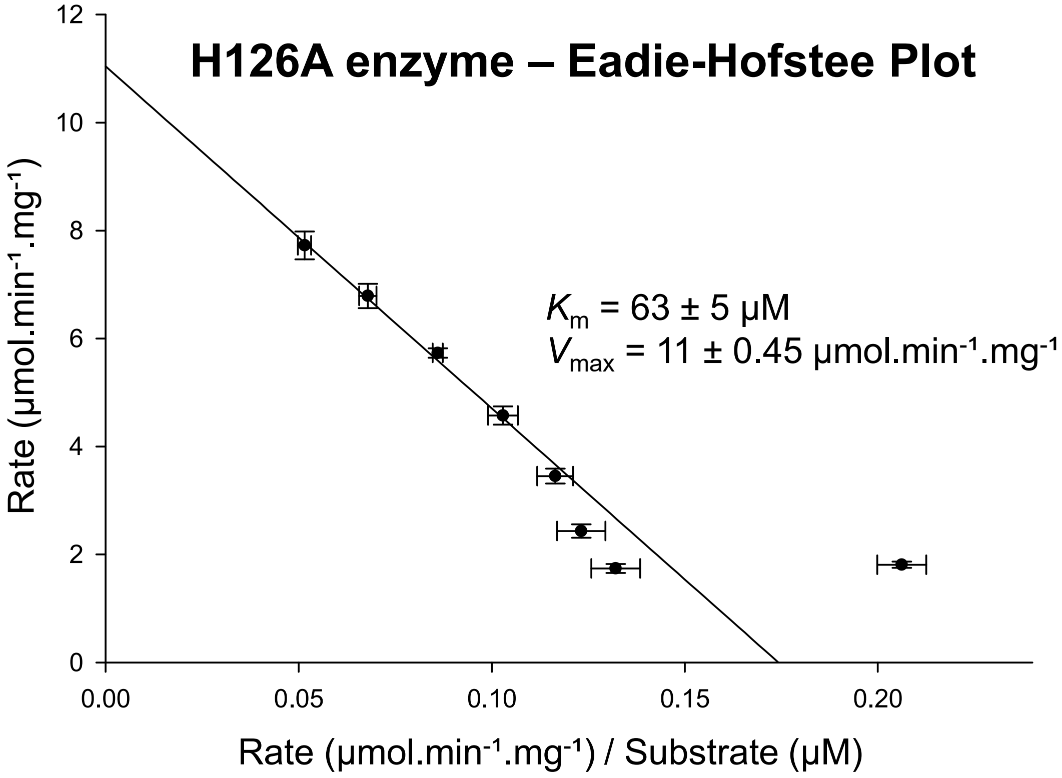
H126A enzyme – Eadie-Hofstee Plot
K_m = 63 ± 5 μM
V_{max} = 11 ± 0.45 μmol.min⁻¹.mg⁻¹
Rate (μmol.min⁻¹.mg⁻¹)
Rate (μmol.min⁻¹.mg⁻¹) / Substrate (μM)
0
2
4
6
8
10
12
0.00
0.05
0.10
0.15
0.20

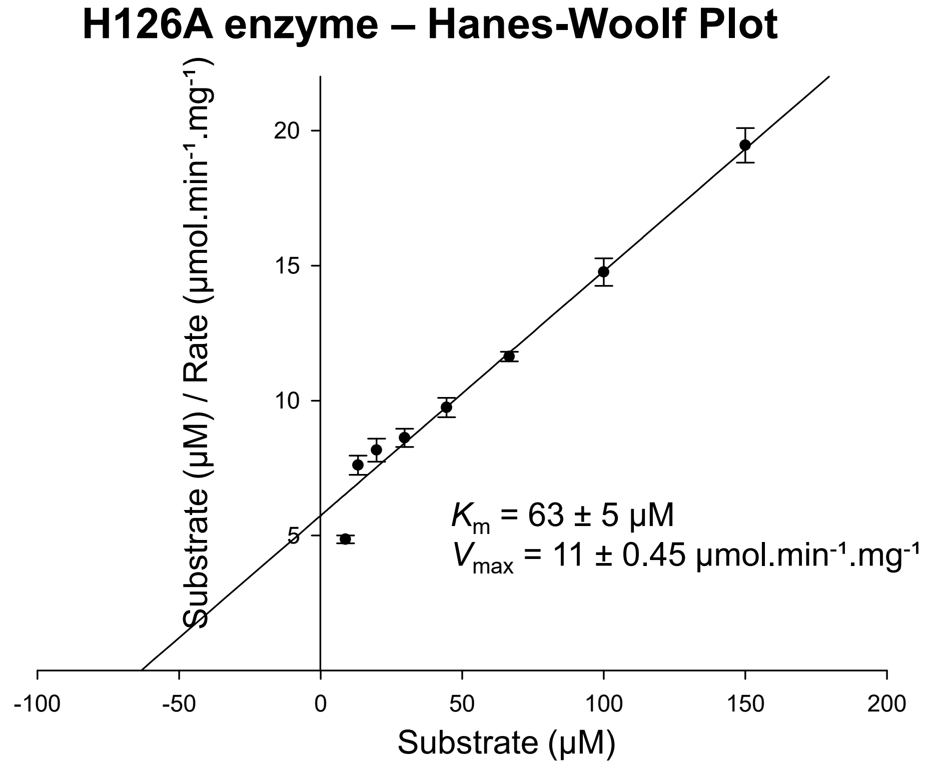
H126A enzyme – Hanes-Woolf Plot
K_m = 63 ± 5 μM
V_{max} = 11 ± 0.45 μmol.min⁻¹.mg⁻¹
Substrate (μM) / Rate (μmol.min⁻¹.mg⁻¹)
Substrate (μM)
5
10
15
20
-100
-50
0
50
100
150
200

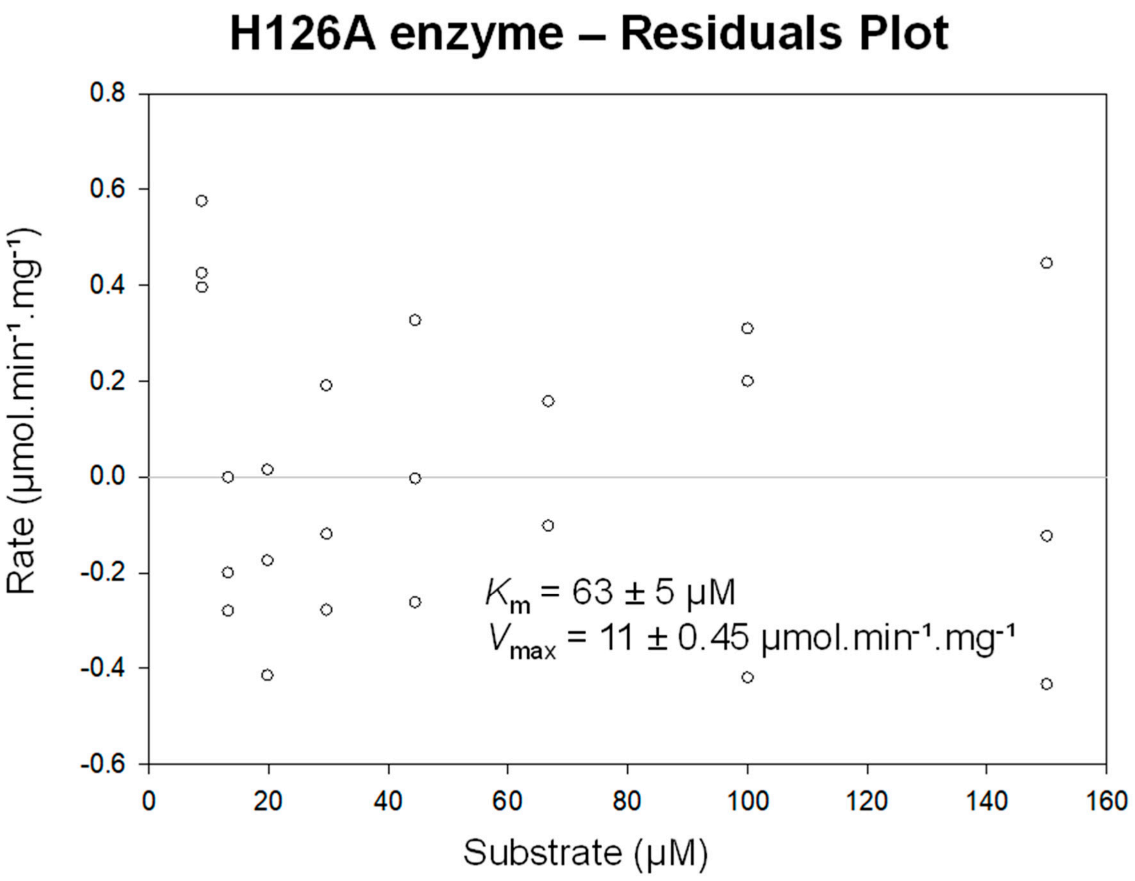

Figure S9. Kinetic data for H126A MCR. Using optimized enzyme concentrations, dilutions of the colorimetric substrate were separately mixed with 1.5 µg/mL H126A MCR and A_{354} data was recorded. This absorbance data was used to determine rates (in µmol.min^{-1}.mg^{-1}). Data for 3 dependent repeats was measured over 10 min. Plotted data are means ± SD.

Figure S10. Kinetic parameters for D156A MCR as determined by the colorimetric assay

Parameters

	Value	± Std. Error	95% Conf. Interval
V_{max} (µmol.min^{-1}.mg^{-1})	47.8515	3.2232	41.1668 to 54.5361
K_m (µM)	70.5296	9.7395	50.3307 to 90.7284

Goodness of Fit

Degrees of Freedom	22
AICc	37.998
R^2	0.962
Sum of Squares	86.600
Sy.x	1.984
Runs Test p Value	0.500

Data

Number of x values	8
Number of replicates	3
Total number of values	24
Number of missing values	0

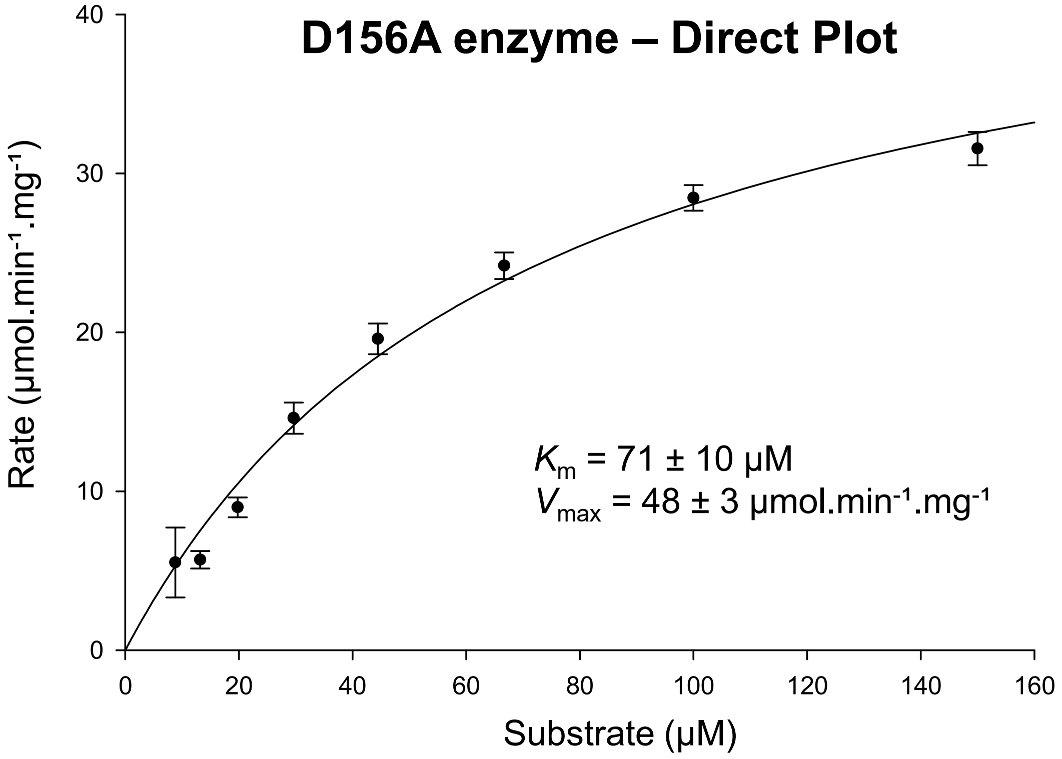

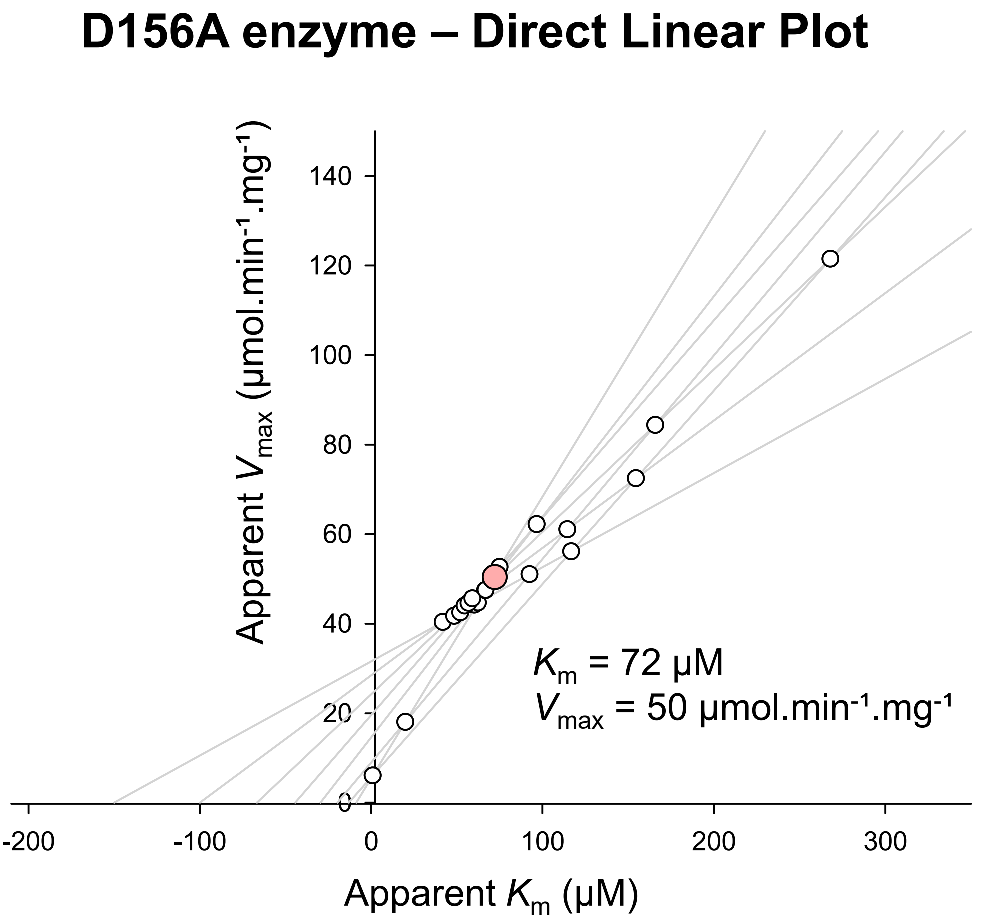
D156A enzyme – Direct Linear Plot
Apparent V_{max} (μmol.min⁻¹.mg⁻¹)
0
20
40
60
80
100
120
140
-200
-100
0
100
200
300
Apparent K_m (μM)
K_m = 72 μM
V_{max} = 50 μmol.min⁻¹.mg⁻¹

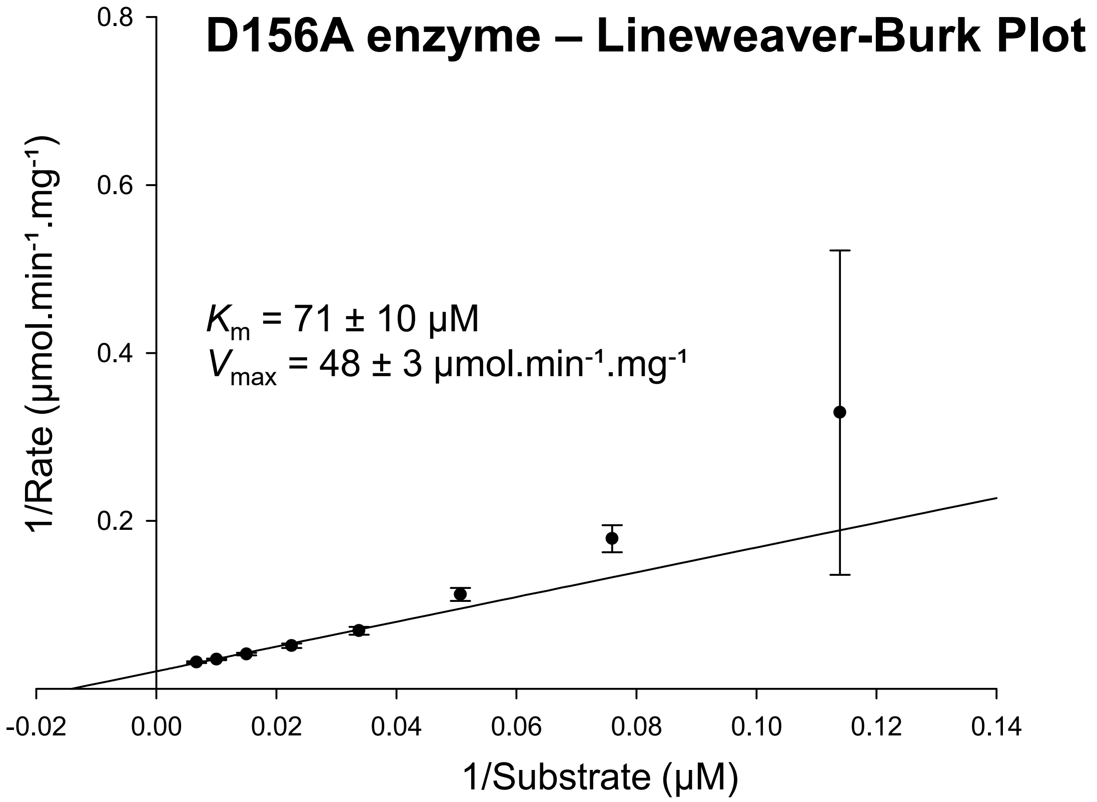
D156A enzyme – Lineweaver-Burk Plot
1/Rate (μmol.min⁻¹.mg⁻¹)
0.2
0.4
0.6
0.8
-0.02
0.00
0.02
0.04
0.06
0.08
0.10
0.12
0.14
1/Substrate (μM)
K_m = 71 ± 10 μM
V_{max} = 48 ± 3 μmol.min⁻¹.mg⁻¹

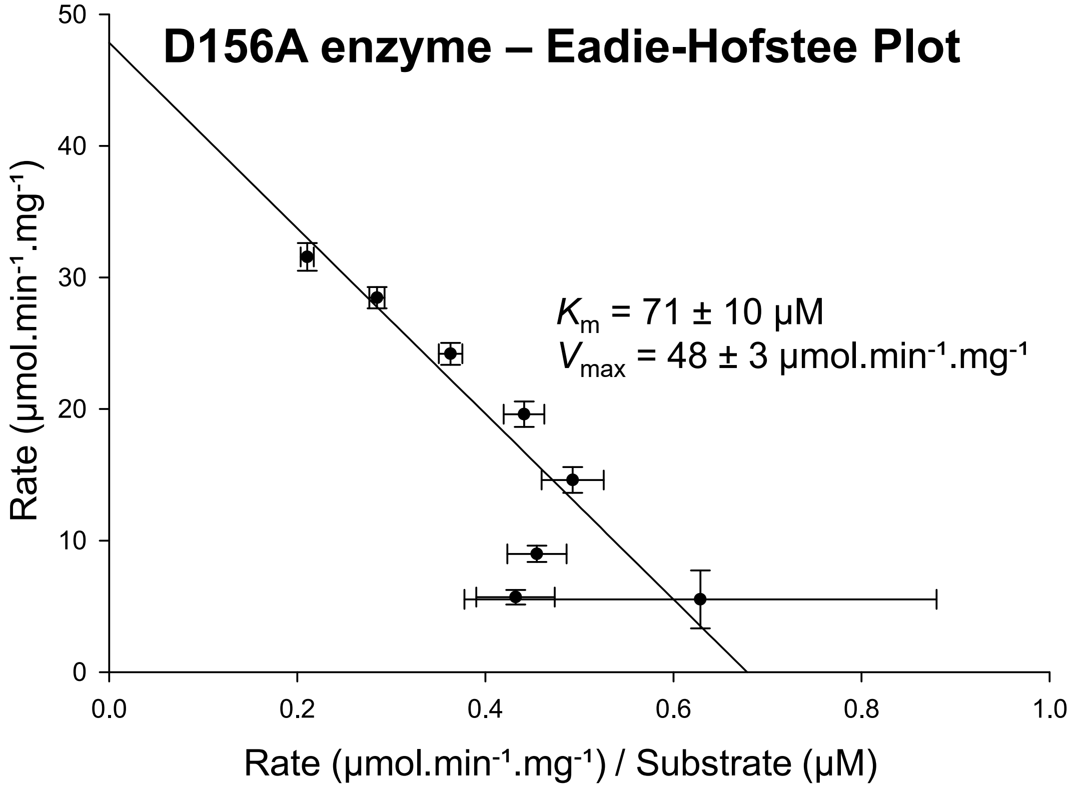
D156A enzyme – Eadie-Hofstee Plot
Rate (µmol.min⁻¹.mg⁻¹)
Rate (µmol.min⁻¹.mg⁻¹) / Substrate (µM)
Km = 71 ± 10 µM
Vmax = 48 ± 3 µmol.min⁻¹.mg⁻¹
0
10
20
30
40
50
0.0
0.2
0.4
0.6
0.8
1.0

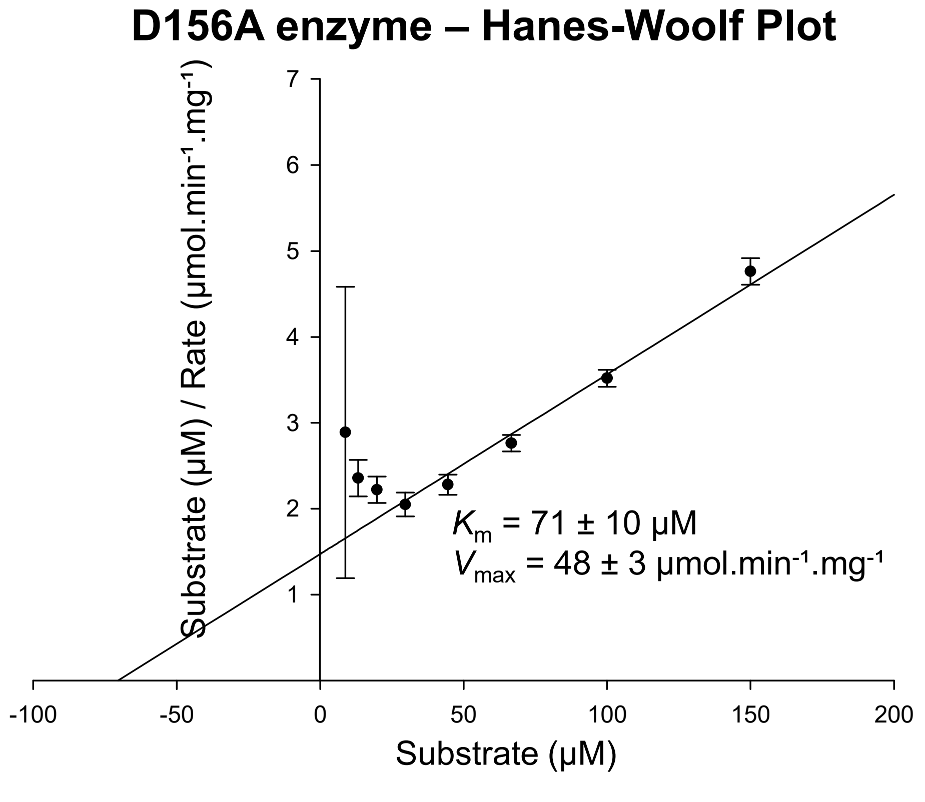
D156A enzyme – Hanes-Woolf Plot
Substrate (µM) / Rate (µmol.min⁻¹.mg⁻¹)
Substrate (µM)
Km = 71 ± 10 µM
Vmax = 48 ± 3 µmol.min⁻¹.mg⁻¹
1
2
3
4
5
6
7
-100
-50
0
50
100
150
200

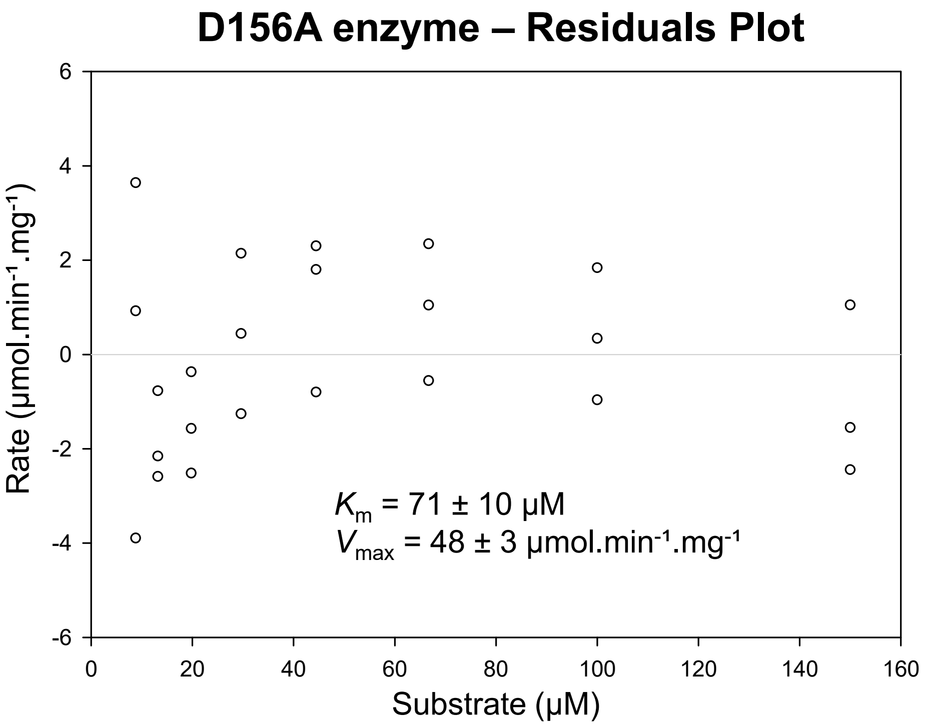

Figure S10. Kinetic data for D156A MCR. Using optimized enzyme concentrations, dilutions of the colorimetric substrate were separately mixed with 0.36 µg/mL H126A MCR and A_{354} data was recorded. This absorbance data was used to determine rates (in µmol.min^{-1}.mg^{-1}). Data for 3 dependent repeats was measured over 10 min. Plotted data are means ± SD.

Figure S11. Kinetic parameters for E241A MCR as determined by the colorimetric assay

Parameters

	Value	± Std. Error	95% Conf. Interval
V_{max} (µmol.min^{-1}.mg^{-1})	23.2168	1.0076	21.1271 to 25.3066
K_m (µM)	24.0352	3.1221	17.5603 to 30.5102

Goodness of Fit

Degrees of Freedom	22
AICc	22.657
R^2	0.915
Sum of Squares	45.699
Sy.x	1.441
Runs Test p Value	0.110

Data

Number of x values	8
Number of replicates	3
Total number of values	24
Number of missing values	0

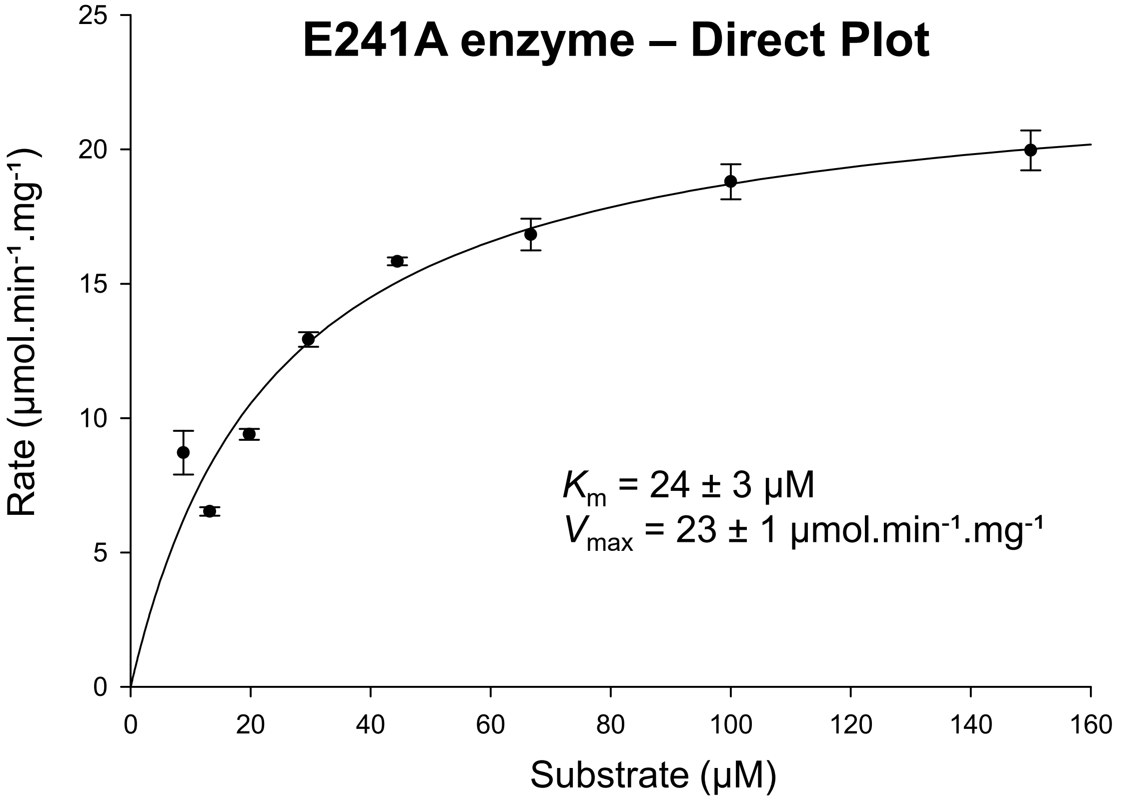
E241A enzyme – Direct Plot
Rate (µmol.min⁻¹.mg⁻¹)
Substrate (µM)
K_m = 24 ± 3 µM
V_{max} = 23 ± 1 µmol.min⁻¹.mg⁻¹
0
5
10
15
20
25
0
20
40
60
80
100
120
140
160

E241A enzyme – Direct Linear Plot

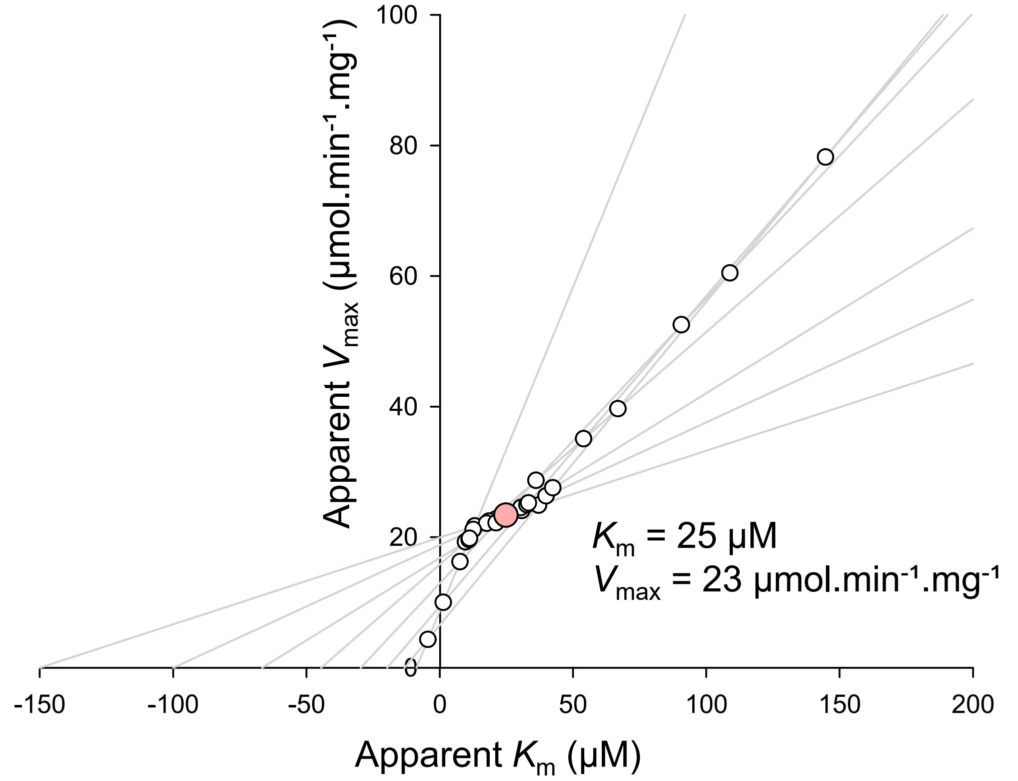

E241A enzyme – Lineweaver-Burk Plot

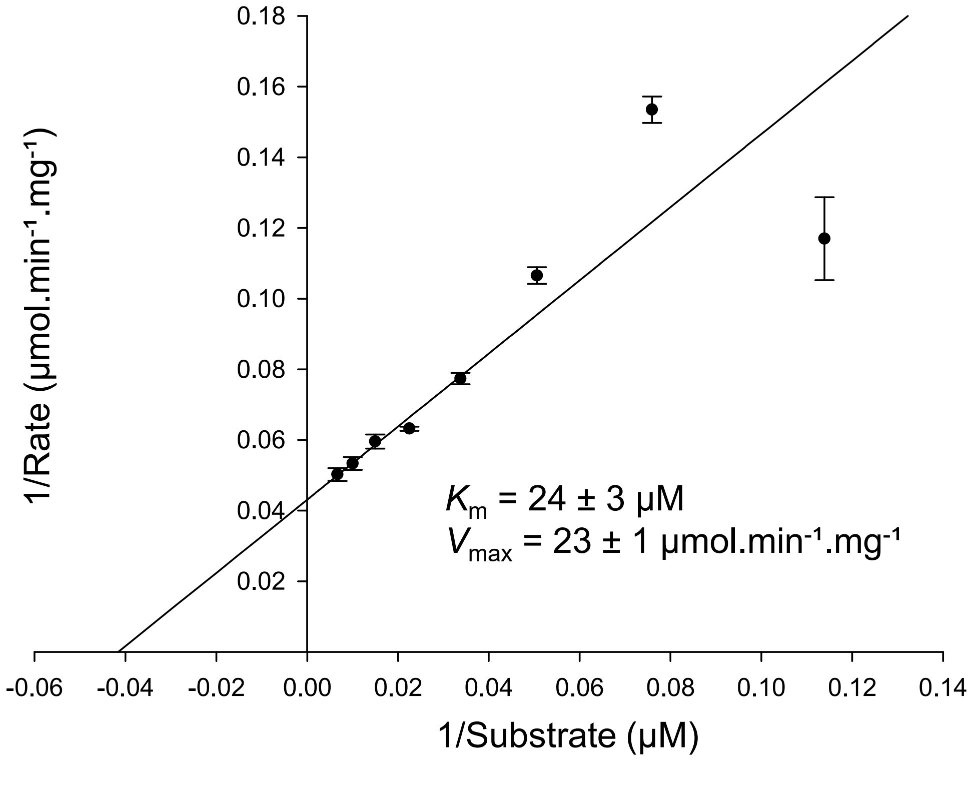

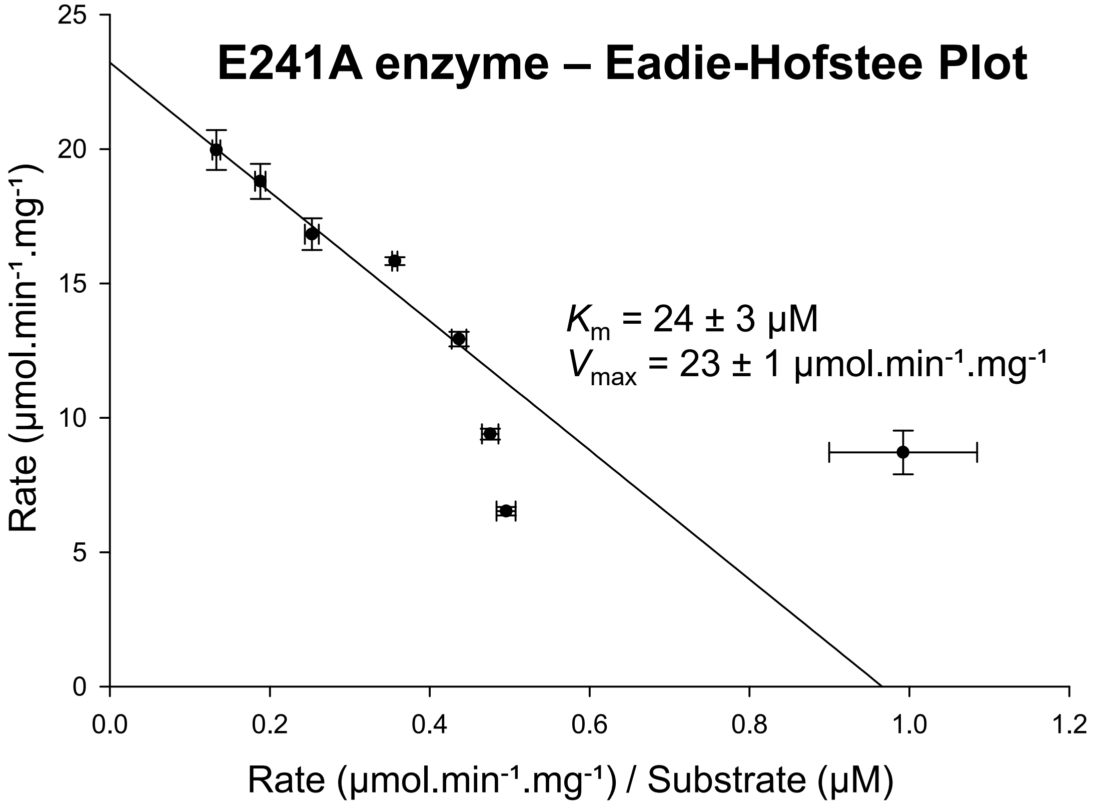
E241A enzyme – Eadie-Hofstee Plot
Km = 24 ± 3 µM
Vmax = 23 ± 1 µmol.min-1.mg-1
Rate (µmol.min-1.mg-1)
Rate (µmol.min-1.mg-1) / Substrate (µM)
0
5
10
15
20
25
0.0
0.2
0.4
0.6
0.8
1.0
1.2

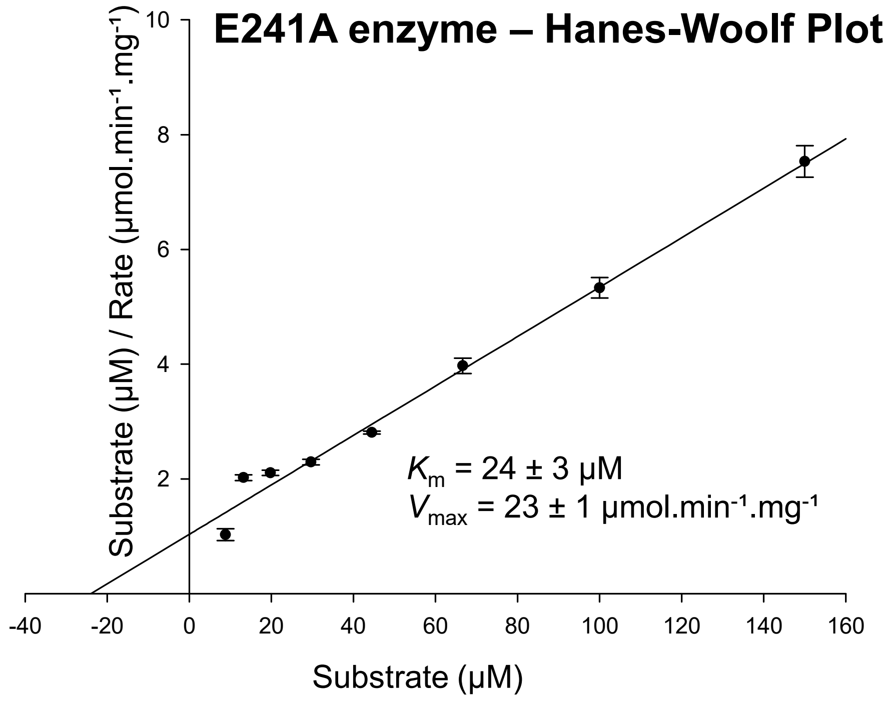
E241A enzyme – Hanes-Woolf Plot
Km = 24 ± 3 µM
Vmax = 23 ± 1 µmol.min-1.mg-1
Substrate (µM) / Rate (µmol.min-1.mg-1)
Substrate (µM)
2
4
6
8
10
-40
-20
0
20
40
60
80
100
120
140
160

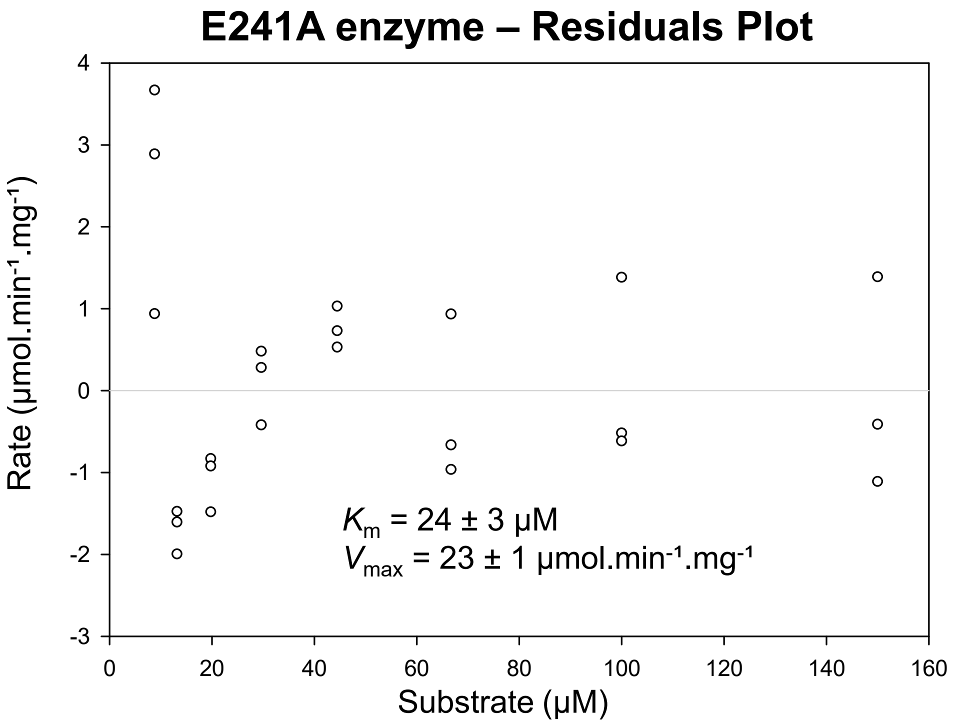

Figure S11. Kinetic data for E241A MCR. Using optimized enzyme concentrations, dilutions of the colorimetric substrate were separately mixed with 0.36 µg/mL H126A MCR and A_{354} data was recorded. This absorbance data was used to determine rates (in µmol.min^{-1}.mg^{-1}). Data for 3 dependent repeats was measured over 10 min. Plotted data are means ± SD.

Table S1. Apparent kinetic parameters of wild-type MCR and its 3 mutants

Enzyme	$K_{m\ (apparent)}$ (µM)	$V_{max\ (apparent)}$ (µmol.min.$^{-1}$mg^{-1})	$k_{cat\ app}$ (s^{-1})	$k_{cat}/K_{m\ app}$ ($M^{-1}.s^{-1}$)
Wild-type MCR	96	162	106	1.1×10^6
H126A MCR	69	11.5	7.5	0.11×10^6
D156A MCR	72	50	33	0.45×10^6
E241A MCR	25	23	15	0.61×10^6
Human AMACR 1A [1]	58	0.112	0.088	1517

Table S1. Apparent kinetic paraments of wild-type MCR and its 3 mutants. The apparent kinetic parameters are derived from fitting colorimetric assay data to the Direct Linear plot.

References

1. Yevglevskis, M.; Lee, G.L.; Nathubhai, A.; Petrova, Y.D.; James, T.D.; Threadgill, M.D.; Woodman, T.J.; Lloyd, M.D. A novel colorimetric assay for α-methylacyl-CoA racemase 1A (AMACR; P504S) utilizing the elimination of 2,4-dinitrophenolate. *Chem. Commun. (Camb.).* **2017**, *53*, 5087-5090, doi: 10.1039/c7cc00476a.
